# The effect of cooling process on the structure and charge/discharge capacities of Li-rich solid-solution layered oxide cathode materials for the Li-ion battery†

**DOI:** 10.1039/d0ra06680j

**Published:** 2021-01-06

**Authors:** Fumihiro Nomura, Tatsuya Watanabe, Hiroya Ochiai, Takao Gunji, Takeshi Hagiwara, Jianfei Wu, Futoshi Matsumoto

**Affiliations:** Department of Materials and Life Chemistry, Kanagawa University 3-27-1, Rokkakubashi, Kanagawa-ku Yokohama Kanagawa 221-8686 Japan fmatsumoto@kanagawa-u.ac.jp; Research Institute for Engineering, Kanagawa University 3-27-1, Rokkakubashi, Kanagawa-ku Yokohama Kanagawa 221-8686 Japan; Qingdao Industrial Energy Storage Research Institute, Qingdao Institute of Bioenergy and Bioprocess Technology, Chinese Academy of Sciences No. 189 Songling Road 266101 Qingdao China

## Abstract

The effect of cooling process after calcination at 900 °C in the preparation of cathode materials, on the crystal structure and charging/discharging capacities of Li_2_MnO_3_–LiNi_1/2_Mn_1/2_O_2_–LiNi_1/3_Mn_1/3_Co_1/3_O_2_ Li-rich solid-solution layered oxide (LLO) cathode materials for the lithium ion battery was examined in twenty-one LLO samples having different compositions. This was achieved by applying two types of cooling processes: (i) quenching the calcinated LLO samples with liquid nitrogen (quenched cooling), and (ii) slow cooling of LLO samples in the furnace at a controlled decreasing rate of the temperature (slow cooling). The results of the comparison between discharging capacities observed with LLO samples prepared with two types of cooling processes indicated that the cooling process for LLO samples to exhibit high discharge capacity was not limited to either one. The process that can be more effective for LLO samples to exhibit the high discharge capacity depended on the composition of LLO samples. LLO samples containing Li_2_MnO_3_ of over 60% exhibited higher discharge capacity when samples were quenched with liquid nitrogen than those prepared with the slow cooling process. Among LLOs examined, the effect of quenching was maximum when the Li_2_MnO_3_ content was 60%. As the LLO composition deviated from the line of 60% Li_2_MnO_3_ in the Li[Li_0.20_Mn_0.58_Ni_0.18_Co_0.04_]O_2_ sample compositions, the effect of quenching became smaller and the slow cooling process was superior to the quenching process. A connection was thus made between the structural difference of LLO samples prepared with the two types of cooling processes and the cathode performance was observed.

## Abstract 2

The difference in discharge capacity = (discharge capacity of the LLO sample prepared by quenched cooling) − (discharge capacity of the LLO sample prepared by slow cooling). The difference in discharge capacity: > 10 mA h g^−1^, −10 mA h g^−1^ < <10 mA h g^−1^,  < −10 mA h g^−1^.

## Graphical abstract

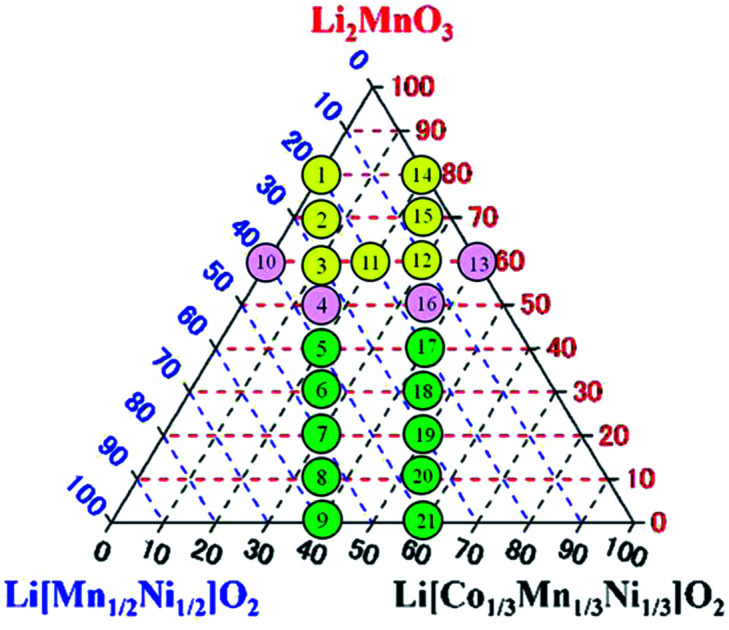

## 1. Introduction

Li_2_MnO_3_–LiMO_2_ (M: transition metal) Li-rich solid-solution layered oxide (LLO) materials have attracted much attention from researchers and engineers in the area of lithium ion batteries (LIBs) because of their high charging/discharging capacities over 250 mA h g^−1^ when LLO materials were charged to more than 4.6 V (*vs.* Li/Li^+^).^1,2^ Although many researchers have made efforts toward elucidating the reason why the LLO materials exhibited high charging/discharging capacities,^3,4^ the mechanism has not been made clear yet. In addition, their capacities degrade with increasing charging/discharging cycling number.^5,6^ A prevention method for the degradation of the cathode performance is also not known to have been established yet. The answers of how to prepare LLO materials exhibiting high and stable capacities is still pursued. In order to establish knowledge on LLO materials from a multilateral point of view, in the LLO samples composed of Li_2_MnO_3_–LiNi_1/2_Mn_1/2_O_2_–LiNi_1/3_Mn_1/3_Co_1/3_O_2_, we have also examined the best ratios of Li_2_MnO_3_, LiNi_1/2_Mn_1/2_O_2_ and LiNi_1/3_Mn_1/3_Co_1/3_O_2_, the calcination temperature and the cooling process for LLO exhibiting the best cathode performance.^7–10^ Among these results, it was found that Li[Li_0.20_Mn_0.58_Ni_0.18_Co_0.04_]O_2_ (Li_2_MnO_3_ (60%)–LiNi_0.5_Mn_0.5_O_2_ (30%)–LiNi_1/3_Co_1/3_Mn_1/3_O_2_ (10%)) exhibited the best cathode performance among LLOs examined, and the cooling process was one of the key factors in determining the cathode performance.^10^ Many papers have reported the use of the quenching process, in which LLO samples calcinated at 800–100 °C were suddenly cooled with liquid nitrogen; thus, the importance of quenching process in achieving LLO samples exhibiting high charging/discharging capacities has been suggested.^11–14^ The difference in the crystal structures between LLO samples prepared with the quenched cooling process, in which calcinated LLO samples were quenched with liquid nitrogen, and the slow cooling process, in which calcinated samples were cooled in the furnace at a controlled decreasing temperature rate, have been discussed.^12,15^ We also have confirmed the necessity of the quenching process for the LLO samples having the composition of Li[Li_0.20_Mn_0.58_Ni_0.18_Co_0.04_]O_2_ (Li_2_MnO_3_ (55%)–LiNi_0.5_Mn_0.5_O_2_ (35%)–LiNi_1/3_Co_1/3_Mn_1/3_O_2_ (10%)).^10^ In addition, quenching the LLO samples decreased the degree of stacking defaults and disordering between the Li^+^ and transition metal ions on the transition metal layer in the crystal structure of the LLO samples, resulting in an improvement of the cathode performance. Slow cooling of the LLO samples degraded the degree of the crystal structure of the LLOs, and reduced the charging/discharging cycle performance.^10^ On the contrary, in the synthesis of layered lithium transition-metal oxides, LiMO_2_ (M = Co, Ni, Mn), quenched cooling was not applied for the LiMO_2_ samples that exhibited high and stable cathode performance.^16,17^ However, there is a paper in which it has been reported that the quenched cooling process is effective in enhancing the cycle stability and storage property in Ni-rich LiMO_2_.^18^ From these papers, we doubt that the application of the cooling processes to cathode materials can be divided into the quenched cooling for the LLO materials and slow cooling for LiMO_2_. So, in this study, we examined the dependence of the charging/discharging capacities on the cooling process in several LLO samples composed of Li_2_MnO_3_–LiNi_1/2_Mn_1/2_O_2_–LiNi_1/3_Mn_1/3_Co_1/3_O_2_. Except for the quenched cooling and slow cooling processes, other cooling processes are considered by changing the cooling rates. In other papers, the quenched cooling and slow cooling processes were used frequently. Therefore, in this study, the quenched cooling and slow cooling processes were examined. It could be confirmed that the necessity of quenched cooling as a cooling process was dependent on the ratio of the Li_2_MnO_3_–LiNi_1/2_Mn_1/2_O_2_–LiNi_1/3_Mn_1/3_Co_1/3_O_2_ in the LLO samples. In addition, what was changed by the difference in the cooling process in the LLO samples was analyzed. Qu *et al.* reported that in the quenched cooling method case, there was an increase in the interslab thickness and decrease in the degree of cation mixing in the LiNi_0.8_Co_0.15_Al_0.05_O_2_ sample.^18^ Chang *et al.* reported an increase in the content of the Li-rich monoclinic component, as well as a larger lattice in the quenched cooling of the Li_1.207_Ni_0.127_Mn_0.54_Co_0.127_O_2_ sample.^14^

In this study, we also gathered the information on the average crystal structure of LLO samples by the Rietveld refinement method with the pXRD patterns and the results of charging/discharging cycle tests, and tried to find the relationship between the cooling process and the cathode performance from the view point of the average crystal structure of the LLO samples with pXRD data.

## 2. Experimental

### 2.1 Preparation of LLO materials

**Fig. 1:**
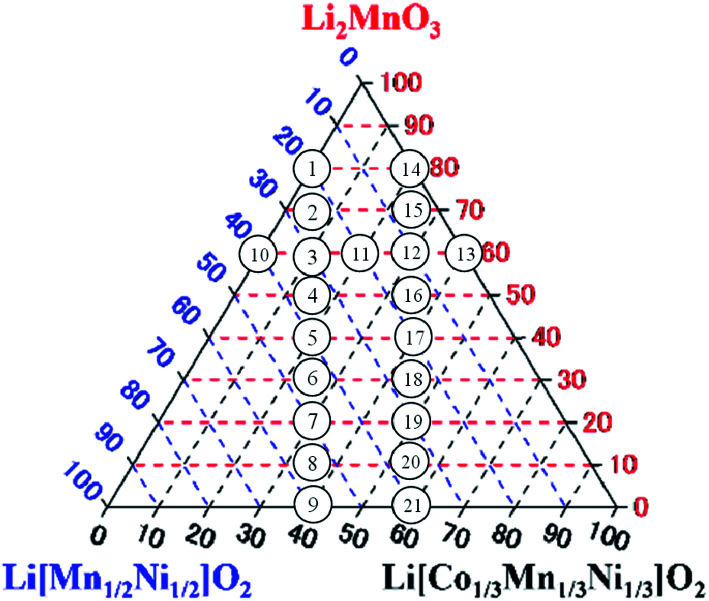
Targeted compositions of synthesized LLO samples represented in the Li_2_MnO_3_–LiNi_1/2_Mn_1/2_O_2_–LiNi_1/3_Mn_1/3_Co_1/3_O_2_ ternary diagram. The numbers 1–21 in the circles indicate the sample number.

The Li_2_MnO_3_–LiNi_1/2_Mn_1/2_O_2_–LiNi_1/3_Mn_1/3_Co_1/3_O_2_ LLO samples examined in this study were synthesized by a co-precipitation procedure using the following commercial reagents: nickel sulfate hexahydrate (NiSO_4_·6H_2_O, 99%, Kanto Chemical. Co., Inc. (Kanto), Japan), manganese sulfate monohydrate (MnSO_4_·H_2_O, 99%, Kanto) and cobalt sulfate heptahydrate (CoSO_4_·7H_2_O, 99%, Wako Pure Chemical Industries, Ltd. (Wako), Japan). All of the reagents were used without further purification. The targeted compositions of twenty-one synthesized LLOs and the ratios of the Li_2_MnO_3_, LiNi_1/2_Mn_1/2_O_2_ and LiNi_1/3_Mn_1/3_Co_1/3_O_2_ components are summarized in Table 1, and are also presented in the Li_2_MnO_3_–LiNi_1/2_Mn_1/2_O_2_–LiNi_1/3_Mn_1/3_Co_1/3_O_2_ ternary diagram of Fig. 1.

**Table 1:** Summary of the targeted compositions of synthesized LLO samples

Sample no.	Targeted compositions of synthesized LLOs	Ratios of Li_2_MnO_3_, LiNi_1/2_Mn_1/2_O_2_ and LiNi_1/3_Mn_1/3_Co_1/3_O_2_ components	Ratios of Mn, Co and Ni in LLOs	Compositions estimated with ICP-MS
1	Li_1.27_Ni_0.1_Mn_0.63_O_2_	80 : 20 : 0	0.864 : 0.000 : 0.136	Li_1.269_Ni_0.096_Mn_0.635_O_2_
2	Li_1.23_Ni_0.14_Mn_0.61_Co_0.02_O_2_	70 : 25 : 5	0.793 : 0.022 : 0.185	Li_1.227_Ni_0.152_Mn_0.616_Co_0.019_O_2_
3	Li_1.2_Ni_0.183_Mn_0.583_Co_0.04_O_2_	60 : 30 : 10	0.729 : 0.042 : 0.229	Li_1.209_Ni_0.173_Mn_0.590_Co_0.028_O_2_
4	Li_1.17_Ni_0.22_Mn_0.56_Co_0.05_O_2_	50 : 35 : 15	0.670 : 0.060 : 0.270	Li_1.168_Ni_0.223_Mn_0.559_Co_0.050_O_2_
5	Li_1.13_Ni_0.27_Mn_0.53_Co_0.07_O_2_	40 : 40 : 20	0.615 : 0.077 : 0.308	Li_1.138_Ni_0.260_Mn_0.537_Co_0.065_O_2_
6	Li_1.1_Ni_0.31_Mn_0.51_Co_0.08_O_2_	30 : 45 : 25	0.565 : 0.093 : 0.343	Li_1.10_Ni_0.310_Mn_0.510_Co_0.08_O_2_
7	Li_1.07_Ni_0.35_Mn_0.48_Co_0.1_O_2_	20 : 50 : 30	0.518 : 0.107 : 0.375	Li_1.063_Ni_0.355_Mn_0.481_Co_0.101_O_2_
8	Li_1.03_Ni_0.39_Mn_0.46_Co_0.12_O_2_	10 : 55 : 35	0.474 : 0.121 : 0.405	Li_1.03_Ni_0.39_Mn_0.46_Co_0.12_O_2_
9	LiNi_0.433_Mn_0.433_Co_0.133_O_2_	0 : 60 : 40	0.433 : 0.133 : 0.433	LiNi_0.433_Mn_0.433_Co_0.133_O_2_
10	Li_1.2_Ni_0.2_Mn_0.6_O_2_	60 : 40 : 0	0.750 : 0.000 : 0.250	Li_1.200_Ni_0.200_Mn_0.600_O_2_
11	Li_1.2_Ni_0.16_Mn_0.57_Co_0.07_O_2_	60 : 20 : 20	0.708 : 0.083 : 0.208	Li_1.207_Ni_0.157_Mn_0.576_Co_0.065_O_2_
12	Li_1.2_Ni_0.15_Mn_0.55_Co_0.1_O_2_	60 : 10 : 30	0.688 : 0.125 : 0.188	Li_1.203_Ni_0.149_Mn_0.550_Co_0.071_O_2_
13	Li_1.2_Ni_0.133_Mn_0.533_Co_0.133_O_2_	60 : 0 : 40	0.667 : 0.167 : 0.167	Li_1.206_Ni_0.139_Mn_0.552_Co_0.103_O_2_
14	Li_1.27_Ni_0.1_Mn_0.63_O_2_	80 : 0 : 20	0.818 : 0.091 : 0.091	Li_1.273_Ni_0.090_Mn_0.637_O_2_
15	Li_1.23_Ni_0.14_Mn_0.61_Co_0.02_O_2_	70 : 5 : 25	0.750 : 0.109 : 0.141	Li_1.231_Ni_0.118_Mn_0.584_Co_0.083_O_2_
16	Li_1.17_Ni_0.22_Mn_0.56_Co_0.05_O_2_	50 : 15 : 35	0.630 : 0.140 : 0.2300	Li_1.204_Ni_0.138_Mn_0.605_Co_0.053_O_2_
17	Li_1.2_Ni_0.27_Mn_0.53_Co_0.07_O_2_	40 : 20 : 40	0.577 : 0.154 : 0.269	Li_1.132_Ni_0.235_Mn_0.499_Co_0.134_O_2_
18	Li_1.2_Ni_0.31_Mn_0.51_Co_0.08_O_2_	30 : 25 : 45	0.528 : 0.167 : 0.306	Li_1.193_Ni_0.312_Mn_0.490_Co_0.05_O_2_
19	Li_1.07_Ni_0.35_Mn_0.48_Co_0.1_O_2_	20 : 30 : 50	0.482 : 0.179 : 0.339	Li_1.065_Ni_0.318_Mn_0.449_Co_0.168_O_2_
20	Li_1.03_Ni_0.39_Mn_0.46_Co_0.12_O_2_	10 : 35 : 55	0.440 : 0.190 : 0.371	Li_1.028_Ni_0.383_Mn_0.458_Co_0.131_O_2_
21	Li_1_Ni_0.43_Mn_0.43_Co_0.14_O_2_	0 : 40 : 60	0.400 : 0.200 : 0.400	Li_1.05_Ni_0.428_Mn_0.430_Co_0.10_O_2_

In this study, selected samples in the ternary diagrams were examined. The points to select the samples were the vertical lines and the horizon line of the composition in the ternary diagram. In the vertical lines, the ratio of LiNi_1/2_Mn_1/2_O_2_ to LiNi_1/3_Mn_1/3_Co_1/3_O_2_ was fixed, and then the ratio of Li_2_MnO_3_ to LiMO_2_ was changed to find the dependence of the cooling process on the ratio of Li_2_MnO_3_ to LiMO_2_. In the horizontal line in the ternary diagram, conversely, the ratio of Li_2_MnO_3_ was fixed, and then the ratio of LiNi_1/2_Mn_1/2_O_2_ to LiNi_1/3_Mn_1/3_Co_1/3_O_2_ was changed to find the dependence of cooling process on the ratio of LiNi_1/2_Mn_1/2_O_2_ to LiNi_1/3_Mn_1/3_Co_1/3_O_2_. The horizon line used in this examination was in the area in which the LLO samples exhibited higher charge/discharge capacity, as mentioned above. We think that an examination of the LLO samples in the two vertical lines and a horizon line was enough to find the relationship between the cooling processes to achieve higher capacity and composition of LLO samples.

The starting material mixture of MnSO_4_·H_2_O, NiSO_4_·6H_2_O and CoSO_4_·7H_2_O with a targeted Ni/MN/Co ratio was added to 100 mL of water, while the total concentration of the transition metal ions in the solution was 2 M. In order to precipitate the transition metal carbonates from the solution, 80 mL of the 2 M sodium carbonate (Na_2_CO_3_, 98%, Wako) solution was injected at the rate of 0.5 cm^3^ s^−1^ into it (kept at 60 °C) with a burette. During the precipitation, its pH was kept at 7.5 by injecting the NH_4_OH solution. The precipitates were filtered off, washed several times with hot water until the filtrate exhibited a neutral pH, and dried under vacuum at 100 °C for 5 h. Lithium carbonate (Li_2_CO_3_, 98%, Kanto) was mixed with the precipitates using a wet planetary ball-milling machine in a Teflon jar (672 mL) containing Teflon balls (diameter 1.5 cm, 88 balls) and acetone (80 mL) at ambient temperature with a speed of 300 rpm for 3 h. To compensate for any possible losses of Li^+^ ions by evaporation of Li during the high temperature calcination, the transition metal carbonates were mixed with an excess (7.0%) of Li_2_CO_3_, in comparison with the amount calculated based on the stoichiometric mole ratios of Li, Ni, Co and Mn in the target LLOs. After drying the mixture at 120 °C for 4 h under vacuum, a pellet (diameter: 2 mm) of the dried mixture was formed using 30 kN of force. The individual pellets were then calcinated at 900 °C for 12 h in air. Two types of cooling processes after calcination were used: one is a quenched cooling process with liquid nitrogen, while another is a slow cooling process at a controlled cooling rate of 25 °C h^−1^ in the furnace from 900 to 25 °C. Hereinafter, the former cooling will be called “quenched cooling” and the latter “slow cooling”. The cooled pellets were ground with a mortar to obtain particles having a surface area of 0.8–0.9 m^2^ g^−1^. The Li : Ni : Co : Mn elemental ratios in the synthesized cathode materials were measured to confirm the target ratios by ICP-MS using an Agilent 7700x spectrometer. The pXRD experiments were performed using CuK_α_ radiation (Rigaku RINT-Ultima III; *λ* = 0.1548 nm) at increments of 0.02 degrees over a range from 20 to 80 degrees. An obliquely finished Si crystal (non-reflection Si plate) was used as a sample holder to minimize the background. Brunauer–Emmett–Teller (BET) surface areas of the cathode particles were measured with a TriStar 3000 surface area instrument (Micromeritic Instrument Corporation). X-ray photoelectron spectroscopy (XPS) measurements (JP-9010 MC, JEOL) were performed to analyze the surface composition of the Mn, Ni and Co ions in the samples prepared, and their bulk composition was analyzed with an X-ray fluorescence spectrometer (XRF, ZSX PrimusIV, Rigaku).

In order to investigate the oxidation states of the Mn, Ni and Co ions of the LLO samples prepared with various compositions and cooling conditions, their X-ray absorption fine structures (XAFS) were analyzed using the BL01B1 beam line at the SPring-8 facility (JASRI, Hyogo, Japan). The Mn, Co and Ni K-edge XAFS spectra were measured using quick XAFS (QXAFS) with transmission mode, and a double-crystal Si (111) monochromator was employed to obtain the XAFS data. The energy levels during the XAFS measurement were calibrated by the pre-edge peak in the spectrum of a Cu foil equal to 8980.3 eV. All XAFS spectra were analyzed using the program package Athena software.^19^

The SEM, XRD and Rietveld refinement analysis data shown in this paper were selected from samples 1, 3, 5, 9, 10 and 13 located in the vertical and horizon lines in the ternary diagram of Fig. 1 due to the space limitation in this paper, and because the comparison of the data obtained from samples 1, 3, 5, 9, 10 and 13 clearly indicates the conclusion of this study. The data of the other samples examined are shown and can be compared in the ESI.†

### 2.2 Cell preparation and electrochemical tests

An accurately weighed quantity of 1 g of each LLO cathode material, 0.121 g of acetylene black (AB, DENKA BLACK, Denki Kagaku Kogyo Ltd., Japan) and 0.084 g of polyvinylidene difluoride (PVdF, KF polymer #9130, 13 wt% in *N*-methyl-2-pyrrolidone (NMP), Kureha, Japan) were mixed in NMP (anhydrous 99.5%, Sigma-Aldrich) using planetary mixing equipment (Mazerustar, KK-250S, KURABO, Japan) to form a homogenous mixture. Each mixture (the solid content is 30–40 wt%) had a suitable viscosity for coating it as the cathode film, while preserving the weight% of LLO : AB : PVdF = 83 : 10 : 7 in the prepared cathode film. The mixture was coated using a doctor-blade coater (100 μm gap) on an aluminum (Al) current collector. The thin film mixture on the Al was dried at 130 °C for 5 h in a vacuum drying oven. The thus-prepared electrodes were pressed with a roll press machine. The loading amount of each LLO on the Al current collector was 2.5–3.0 mg cm^−2^. Each prepared cathode layer was kept at the loading density of 1.6 mg cm^−3^.

The cathode performance tests were performed using a CR2032 coin-type cell. The test cells were composed of a cathode and a lithium metal anode separated by a porous polypropylene film (Celgard 3401). The electrolyte used was a 1 M lithium hexafluorophosphate (LiPF_6_)–ethylene carbonate (EC)/dimethyl carbonate (DMC) mixture (1 : 2 by vol., Ube Chemicals, Japan). The charge/discharge cycling was performed using a multichannel battery tester (HJ1001SD8, Hokuto Denko Corporation, Japan). All tests were performed in a constant temperature bath (MIR-154, Panasonic) of 25 °C. The constant-current/constant-voltage (CC-CV) mode was used for the cycling tests. The charge/discharge cycling tests after electrochemical pre-treatment process were performed at a charge/discharge current density of 0.1C-rate. To observe the stable charge/discharge cycle performance, the pre-treatment process developed by Ito *et al.*^20,21^ was applied in this study. The discharge voltage limit was fixed at 2.0 V, and the charge voltage limit was increased by 0.1 V stepwise from 4.5 to 4.8 V every two cycles. The C-rate was calculated with the specific capacity of 200 mA h g^−1^ for all synthesized LLO materials. The cutoff voltages for the charging/discharging tests were 2.0 and 4.8 V. During CV mode, the upper cutoff voltage was held constant for 5 h.

## 3. Results and discussion

### 3.1 Characterization of the synthesized LLO cathode materials and charging/discharging cycle tests of the LLO samples

**Fig. 2:**
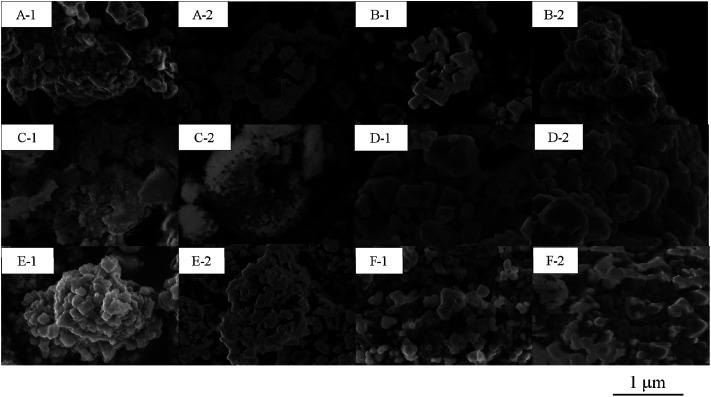
SEM images of the LLO samples prepared by (A-1, B-1, C-1, D-1, E-1 and F-1) quenching the samples calcinated at 900 °C for 12 h in air with liquid nitrogen, and (A-2, B-2, C-2, D-2, E-2 and F-2) cooling them at a controlled cooling rate of 25 °C h^−1^ in the furnace from 900 to 25 °C. (A-1, A-2): sample 1, (B-1, B-2): sample 3, (C-1, C-2): sample 5, (D-1, D-2): sample 9, (E-1, E-2): sample 10 and (F-1, F-2): sample 13.

The synthesized LLO samples had the targeted compositions with an error of 15%, which were evaluated with ICP-MS, as shown in Table 1, and the BET surface areas of 0.8–0.9 m^2^ g^−1^. All secondary particles of the LLO samples were constructed with primary particles having the diameter of 100–200 nm, as shown in Fig. 2 and S1.† From these results, the synthesized samples almost have the same morphology of particles between the quenched and slow cooling processes, and their compositions were changed systematically as we designed. In order to know the deviation of Mn, Ni and Co ions in the sample particles, the surface and bulk compositions of the samples were analyzed with XPS and XRF, respectively (Table S1†). The bulk composition evaluated with XRF (Table S1†) was in good agreement with the targeted composition (Table 1) on each sample. In all samples irrespective of quenched and slow cooling processes, Ni atom is rich in the particle surface areas when compared with bulk. Conversely, the Mn atom is poor in the surface area when compared with the results of the bulk atomic and surface ratios in Table S1.† Although the surface composition of the Co atom is rich or poor in the sample surfaces, the reason why the trend of the composition of Co could not be observed is due to the low content of Co atom in the LLO samples. The degree of Ni richness in the particle surface increases a little when the slow cooling process was applied to the samples in comparison with one prepared using the quenched cooling process. The degree of Ni richness in the slow cooling process does not depend on the bulk composition. That means that the degree of difference in the composition between the surface and bulk areas was not caused by the difference of the cooling process applied when the samples were prepared.

**Fig. 3:**
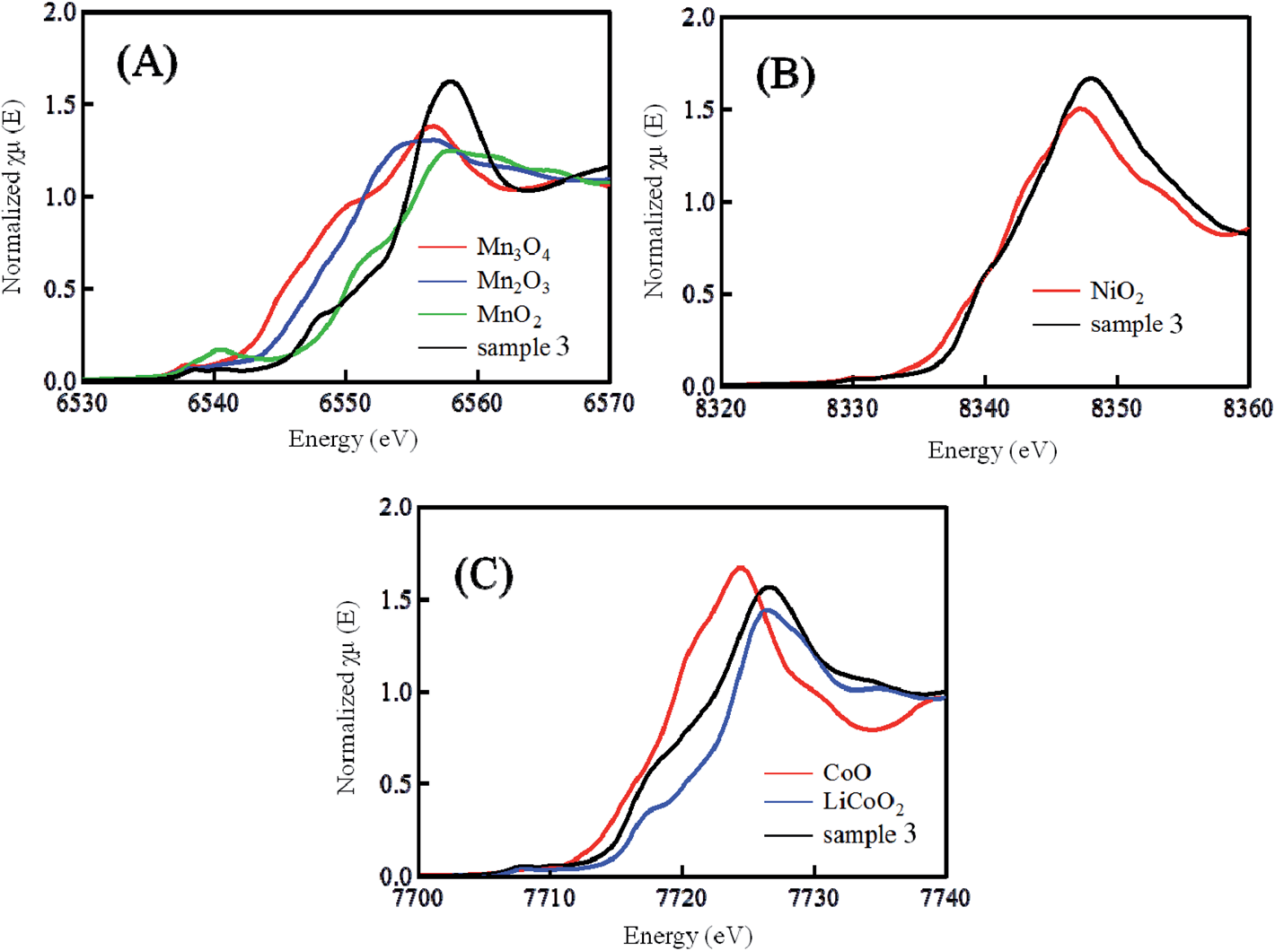
(A) Mn, (B) Ni and (C) Co K-edge XANES spectra of sample 3 prepared by the quenched cooling with liquid nitrogen and reference samples of Mn_3_O_4_, Mn_2_O_3_, MnO, NiO_2_, CoO and LiCoO_2_.

Fig. 3 shows the (A) Mn, (B) Ni and (C) Co K-edge XANES spectra of sample 3 prepared by quenching the calcinated sample with liquid N_2_. Together with the spectra of sample 3, the spectra of the reference samples of Mn_3_O_4_, Mn_2_O_3_, MnO_2_, NiO, CoO, LiCoO_2_ are also shown in Fig. 3. Comparing the sample spectra with the reference spectra, the oxidation number of Mn ion in the sample 3 might be close to +4 because the sample spectra are similar to those of MnO_2_. From a similar point of view, the oxidation number of the Ni and Co ions might be +2 and +3. These oxidation numbers of the Mn, Ni and Co ions agree with ones reported in published papers.^22–24^ Fig. S2† shows the difference in the K-edge XANES spectra of the Mn, Ni and Co ions between the samples 2–8 prepared by quenching the calcinated sample with liquid N_2_ (black line), and low cooling of the calcinated samples at the controlled rate of 25 °C h^−1^ (red line). As mentioned in Fig. 3, the slope of the K-edge XANES spectra depended on the oxidation states of the Mn, Ni and Co ions in the LLO samples.^25^ In the XANES spectra shown in Fig. S2,† the difference (black *vs.* red lines) in the slopes of the XANES spectra observed with the samples prepared with the quenching and slow cooling processes could not be observed. The XANES spectra of the Co ions in the LLO samples exhibited a small difference between the spectra obtained with the samples prepared with quenching and slow cooling processes. The small difference in the spectra obtained with the samples prepared with the quenching and slow cooling processes is due to the low content of Co ion. These results indicate that the average oxidation states of the Mn, Ni and Co ions in the LLO samples are not affected by changing the cooling process. From all results shown above, the surface morphology, surface composition and the electronic state of ions in the samples are not the factors that exhibit a difference in the discharge capacity of the LLO samples prepared with quenched and slow cooling processes. The difference in the crystal structure of the LLO samples prepared with quenched and slow cooling processes, as shown below, can be discussed to elucidate the effect of the cooling process on the cathode performance.

**Fig. 4:**
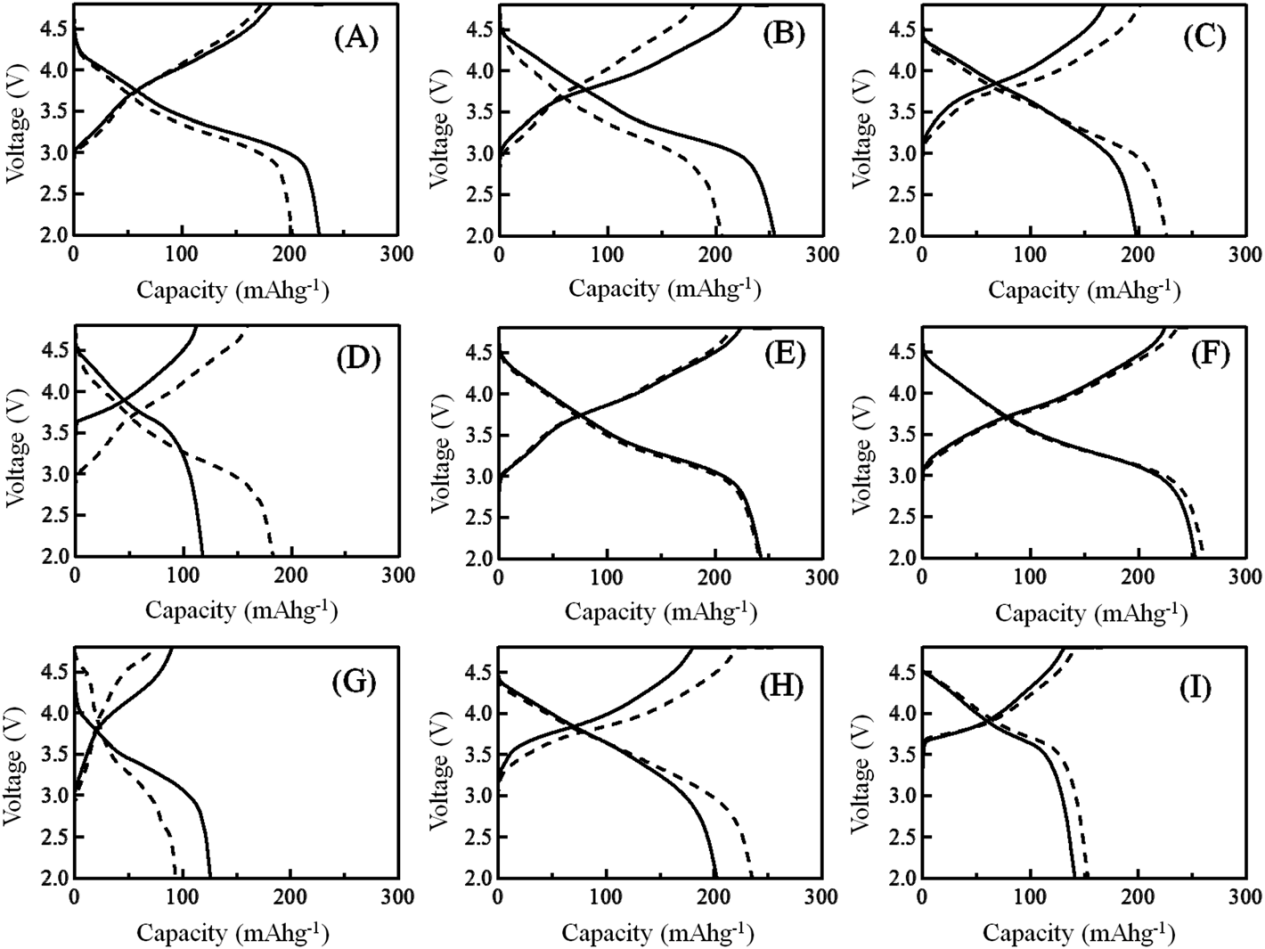
Charging/discharging voltage–capacity curves obtained at the 10^th^ cycle with samples 1 (A), 3 (B), 5 (C), 9 (D), 10 (E), 13 (F), 14 (G), 17 (H) and 21 (I) prepared by the quenched cooling with liquid nitrogen (solid lines), and the slow cooling in the furnace at a controlled rate of 25 °C h^−1^ (dotted lines). The charging/discharging rate was 0.1C.

**Fig. 5:**
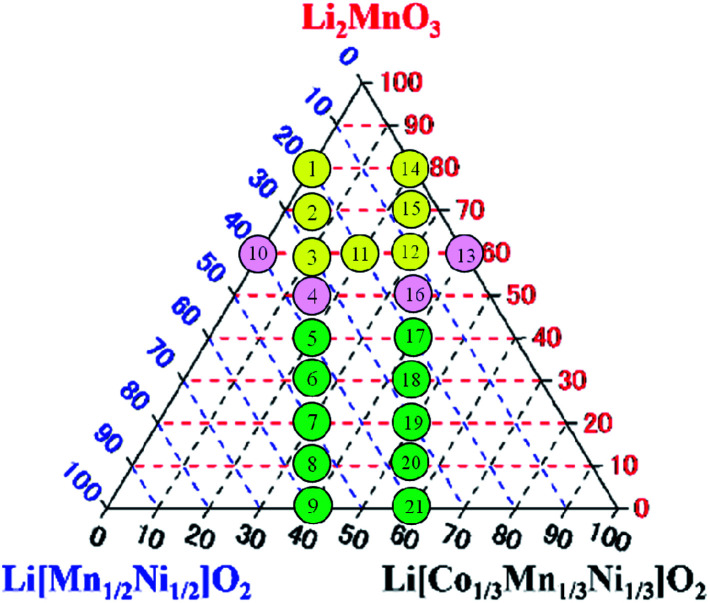
Summary of the dependence of the difference in discharge capacity obtained at the 10^th^ cycle on the targeted composition of the LLO samples. The difference in discharge capacity = (discharge capacity of the LLO sample prepared by the quenched cooling) − (discharge capacity of the LLO sample prepared by slow cooling). The difference in discharge capacities: 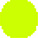 > 10 mA h g^−1^, −10 mA h g^−1^ < 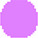 < 10 mA h g^−1^, 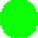 < −10 mA h g^−1^.

Fig. 4 shows the charging/discharging voltage–capacity curves observed at the 10^th^ cycle with the samples 1, 3, 5, 9, 10, 13, 14, 17 and 21. The charging/discharging voltage–capacity curves for the other samples are shown in Fig. S3.† In all samples, the charging/discharging voltage–capacity curves exhibited characteristic large voltage hysteresis during the charging and discharging processes, although the conventional LiCoO_2_ and LiNi_1/3_Mn_1/3_Co_1/3_O_2_ cathode materials exhibited almost constant voltages in both charging and discharging processes.^26^ The difference in the shape of the charging/discharging voltage–capacity curves between the samples prepared by quenched and slow cooling processes could not be observed in all LLO samples tested. Depending on the composition of the LLO samples, the observed capacities and the magnitude of the retention of charge/discharge capacities observed were changed. This means that in some compositions, the quenched cooling process was effective for observing the higher capacity. In other compositions, the LLO samples prepared with the slow cooling process exhibited higher capacity. In Fig. S4,† d*Q*/d*V* curves calculated with the charging/discharging capacity *vs.* voltage curves at the 10^th^ cycle are shown. Generally, the difference in the d*Q*/d*V* curves between the samples prepared by quenched and slow cooling processes could be observed in the area where the Li_2_MnO_3_ percentages in the samples are high (samples 1, 2, 3 and 14 and 15) and low (samples 8, 9, 20 and 21). However, the difference in the d*Q*/d*V* curves could not be observed in the area (samples 4, 5, 6, 7, 12, 16, 17 and 18) where the difference in the charging/discharging capacities between the samples prepared by quenching and slow cooling processes could be observed (around 60% Li_2_MnO_3_ composition). Therefore, the difference in the cooling process does not influence the redox potentials of the Mn, Ni and Co ions in the LLO samples. Fig. S5† shows the results of the charging/discharging cycle performance tests obtained at 0.1C. Until the 6^th^ cycle, the pre-treatment explained in the experimental section was applied to all cathodes. From the 7^th^ cycle, the charging/discharging cycle tests were started between the cutoff voltages of 4.8 and 2.0 V. The discharge capacities at the 10^th^ cycle are summarized in Table 2. In the LLO samples 3, 11, 12, 13 that contained the Li_2_MnO_3_ component with high percentage and exhibited higher discharge capacities in the quenched samples, the discharge capacity decreased with increasing cycle number even though the samples exhibited high discharge capacity. These results indicate that the samples exhibiting high discharge capacity do not always exhibit high cycle durability, and that improvement of the cycle durability of the LLO samples cannot be achieved by selecting the cooling process after calcination of the LLO samples. From Table 2, the difference in the discharge capacities observed at the 10^th^ cycle with the samples prepared by quenched and slow cooling processes was calculated, and their difference in the composition of LLO is summarized in Fig. 5. In Fig. 5, the colored circles show the degree of the difference in the discharge capacities between the quenched and slow cooling samples. The difference in the discharge capacities is defined as follows:

1 Difference of the discharge capacity = {(discharge capacity of the LLO sample prepared by quenching) − (discharge capacity of the LLO sample prepared by slow cooling)} at the 10^th^ discharge cycle

**Table 2:** Summary of the discharge capacity of LLO samples (no. 1–21) at the 10^th^ cycle. The samples were prepared by quenching the samples calcinated at 900 °C for 12 h in air with liquid nitrogen (“quenched cooling”), and cooling them at a controlled cooling rate of 25 °C h^−1^ in the furnace from 900 to 25 °C (“slow cooling”). The discharging rate was 0.1C

Sample no.	Discharge capacity (mA h g^−1^)	
	Quenched cooling	Slow cooling
1	227	203
2	286	232
3	260	205
4	234	236
5	198	226
6	150	170
7	167	181
8	197	211
9	172	183
10	233	241
11	264	242
12	254	236
13	253	261
14	133	102
15	176	150
16	188	204
17	200	234
18	186	201
19	153	169
20	168	186
21	141	154

The LLO samples prepared by quenching in the yellow colored circles exhibited higher discharge capacity than those prepared by slow cooling. The samples in the pink area, which adjoined the yellow area, showed that the discharge capacities of the samples prepared with quenching and slow cooling did not have a large difference. In the green circles, the slow cooling process was effective for the samples to display higher discharge capacity. A clear classification of the composition could be observed for the question of which cooling process was effective to observe higher discharge capacity.

### 3.2 Evaluation of the difference of the crystal structure of the LLO samples after quenching and slow cooling processes with pXDR and Rietveld refinement analysis

**Fig. 6:**
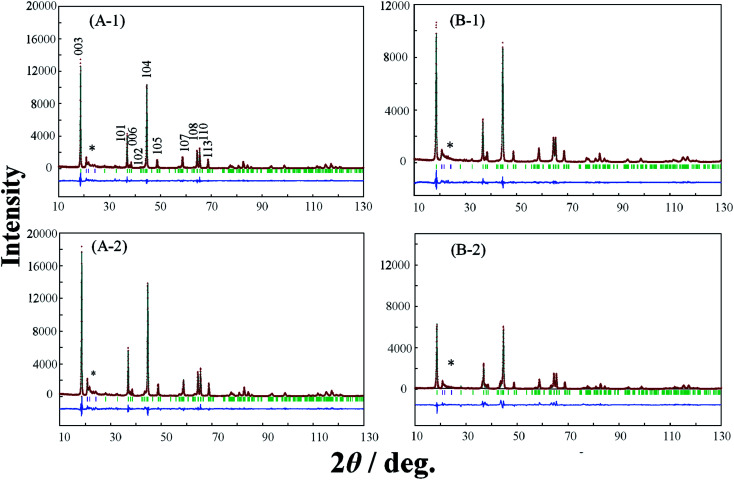
XRD Rietveld refinement profiles of samples 1 (A-1, A-2) and 3 (B-1, B-2) prepared by (A-1, B-1) the quenched cooling with liquid nitrogen, and (A-2, B-2) the slow cooling in the furnace at a controlled rate of 25 °C h^−1^. (—) observed, (—) calculated, (—) the difference of both. The green vertical marks indicate the position of the Bragg reflections. * corresponds to the peaks around 20–25° that result from the ordering of Li^+^ ions in the transition metal layers.

**Fig. 7:**
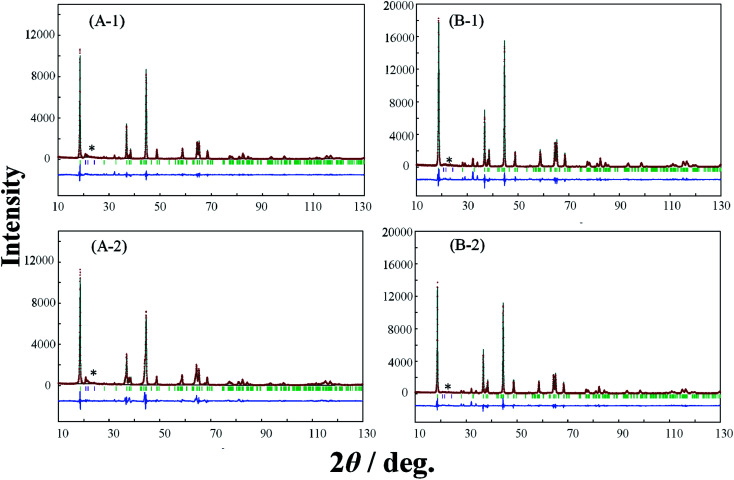
XRD Rietveld refinement profiles of samples 5 (A-1, A-2) and 9 (B-1, B-2) prepared by (A-1, B-1) the quenched cooling with liquid nitrogen, and (A-2, B-2) the slow cooling in the furnace at a controlled rate of 25 °C h^−1^. (—) observed, (—) calculated, (—) the difference of both. The green vertical marks indicate the position of the Bragg reflections. * corresponds to the peaks around 20–25° that result from the ordering of Li^+^ ions in the transition metal layers.

**Fig. 8:**
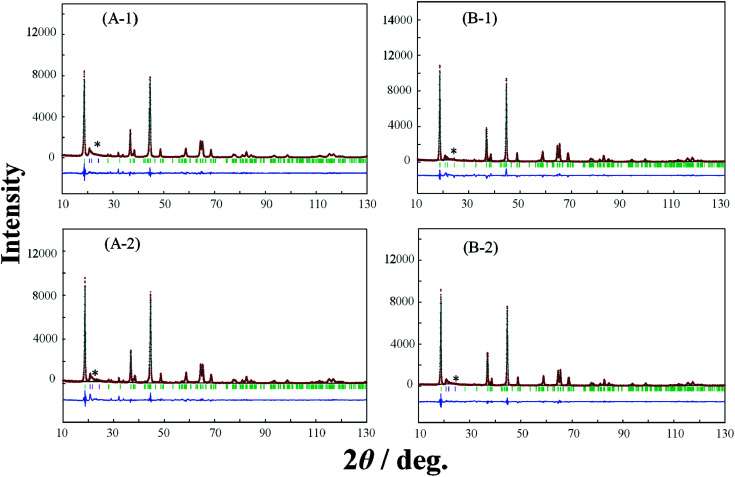
XRD Rietveld refinement profiles of samples 10 (A-1, A-2) and 13 (B-1, B-2) prepared by (A-1, B-1) the quenched cooling with liquid nitrogen, and (A-2, B-2) slow cooling in the furnace at a controlled rate of 25 °C h^−1^. (—) observed, (—) calculated, (—) the residual difference of both. The green vertical marks indicate the position of the Bragg reflections. * corresponds to the peaks around 20–25° that result from the ordering of Li^+^ ions in the transition metal layers.

In order to elucidate the difference of the crystal structure of the LLO samples prepared with the two types of cooling processes, pXRD patterns of the LLO samples prepared with the two types of cooling processes were obtained. Typical pXRD patterns of samples 1, 3, 5, 9, 10 and 13 are shown in Fig. 6–8, and other results are shown in Fig. S6.† The pXRD Rietveld refinement profiles of the LLO samples are also shown as calculated profiles and the residual difference in Fig. 6–8 and S6.†

In Table 3 and S2,† the refined structural parameters of the LLO samples obtained from the Rietveld refinement^21,27^ with the space group of *C*2/*m* are summarized. The comparison of the structural parameters obtained is summarized in Table 4. The definition of the colors used in Table 4 is the same as those used for the difference in the discharge capacity between the samples prepared by quenching and slow cooling in Fig. 5. In all XRD patterns, the peaks at around 20–25° that result from the ordering of Li^+^ ions in the transition metal layers could be observed.^28^ Considering the composition of samples 9 and 21, the samples do not contain the component of Li_2_MnO_3_. Therefore, the sample should have a layered α-NaFeO_2_-type rock salt structure (LiMO_2_) with the space group *R*3̄*m*. However, in this study, the XRD profiles observed with samples 9 and 21 prepared with the two cooling processes exhibited the peaks at around 20–25°. This indicates the ordering of Li^+^ ions in the transition metal layers as a result of cation mixing between the transition metal ions in the transition layer, and Li^+^ ions in the Li^+^ ion layer in the layered α-NaFeO_2_-type rock salt structure. The appearance of the peaks might result from the synthesis condition for this study. The results of the Rietveld refinement analysis demonstrate excellent fitting between the observed Rietveld refinement profile and the calculated one with a *C*2/*m* space group. In particular, the consistency between the XRD data and simulated pattern around 20–25° could be seen in the entire Rietveld refinement, leading to reliable consequences about the Li/Ni mixing degree (Fig. S7†).

The refined structural parameters of LLO samples obtained from the Rietveld refinement with the space of *C*2/*m*. The LLO samples were prepared by (1) the quenched cooling with liquid nitrogen, and (2) the slow cooling in the furnace at a controlled rate of 25 °C h^−1^. Samples: 1 (A), 3 (B), 5 (C), 9 (D), 10 (E) and 13 (F)

**Table:** 

(A-1)					
*S* = 1.9821, *R*_B_ = 4.383, *R*_F_ = 3.712, *R*_wp_ = 13.511					
*a* = 4.9385(3) Å, *b* = 8.5551(4) Å, *c* = 5.0326(2) Å, *β* = 108.997(7)°					
Atom	Site	*x*	*y*	*z*	Occ_-refined_
Li	2c	0	0	0.5	0.975(2)
M	2c	0	0	0.5	0.025(2)
Li	2b	0	0.5	0	0.748(6)
M	2b	0	0.5	0	0.252(6)
Li	4g	0	0.1646(3)	0	0.040(6)
M	4g	0	0.1646(3)	0	0.960(6)
Li	4h	0	0.681(2)	0.5	0.996(1)
M	4h	0	0.681(2)	0.5	0.004(1)
O	4i	0.213(2)	0	0.2169(9)	1
O	8j	0.257(1)	0.3151(6)	0.2270(6)	1

**Table:** 

(A-2)					
*S* = 2.0339, *R*_B_ = 6.018, *R*_F_ = 5.440, *R*_wp_ = 12.940					
*a* = 4.9341(3) Å, *b* = 8.5419(3) Å, *c* = 5.0343(2) Å, *β* = 109.202(4)°					
Atom	Site	*x*	*y*	*z*	Occ_-refined_
Li	2c	0	0	0.5	0.954(3)
M	2c	0	0	0.5	0.046(3)
Li	2b	0	0.5	0	0.546(5)
M	2b	0	0.5	0	0.454(5)
Li	4g	0	0.1697(3)	0	0.054(5)
M	4g	0	0.1697(3)	0	0.946(5)
Li	4h	0	0.699(2)	0.5	0.998(2)
M	4h	0	0.699(2)	0.5	0.002(2)
O	4i	0.208(1)	0	0.220(1)	1
O	8j	0.261(1)	0.3209(7)	0.2195(7)	1

**Table:** 

(B-1)					
*S* = 1.7132, *R*_B_ = 4.117, *R*_F_ = 3.246, *R*_wp_ = 11.975					
*a* = 4.9502(5) Å, *b* = 8.5725(6) Å, *c* = 5.0247(3) Å, *β* = 109.02(7)°					
Atom	Site	*x*	*y*	*z*	Occ_-refined_
Li	2c	0	0	0.5	0.982(2)
M	2c	0	0	0.5	0.018(2)
Li	2b	0	0.5	0	0.605(5)
M	2b	0	0.5	0	0.395(5)
Li	4g	0	0.1642(2)	0	0.031(5)
M	4g	0	0.1624(2)	0	0.969(5)
Li	4h	0	0.681(1)	0.5	0.978(1)
M	4h	0	0.681(1)	0.5	0.022(1)
O	4i	0.217(2)	0	0.213(1)	1
O	8j	0.263(1)	0.3154(6)	0.2303(7)	1

**Table:** 

(B-2)					
*S* = 2.5756, *R*_B_ = 11.247, *R*_F_ = 7.649, *R*_wp_ = 19.371					
*a* = 4.93366(5) Å, *b* = 8.54179(6) Å, *c* = 5.02738(3) Å, *β* = 109.18(7)°					
Atom	Site	*x*	*y*	*z*	Occ_-refined_
Li	2c	0	0	0.5	0.946(5)
M	2c	0	0	0.5	0.054(5)
Li	2b	0	0.5	0	0.596(5)
M	2b	0	0.5	0	0.404(5)
Li	4g	0	0.1642(2)	0	0.059(5)
M	4g	0	0.1624(2)	0	0.941(7)
Li	4h	0	0.681(1)	0.5	0.970(3)
M	4h	0	0.681(1)	0.5	0.030(1)
O	4i	0.216(1)	0	0.220(1)	1
O	8j	0.265(2)	0.3327(7)	0.2250(7)	1

**Table:** 

(C-1)					
*S* = 1.8172, *R*_B_ = 4.806, *R*_F_ = 5.012, *R*_wp_ = 13.040					
*a* = 4.95601(2) Å, *b* = 8.5612(3) Å, *c* = 5.0164(1) Å, *β* = 109.109(4)°					
Atom	Site	*x*	*y*	*z*	Occ_-refined_
Li	2c	0	0	0.5	0.950(8)
M	2c	0	0	0.5	0.050(8)
Li	2b	0	0.5	0	0.349(6)
M	2b	0	0.5	0	0.651(6)
Li	4g	0	0.1646(3)	0	0.098(6)
M	4g	0	0.1646(3)	0	0.902(6)
Li	4h	0	0.681(1)	0.5	0.973(4)
M	4h	0	0.681(1)	0.5	0.027(4)
O	4i	0.215(2)	0	0.221(2)	1
O	8j	0.269(2)	0.3152(6)	0.227(1)	1

**Table:** 

(C-2)					
*S* = 2.0646, *R*_B_ = 4.020, *R*_F_ = 2.430, *R*_wp_ = 15.57					
*a* = 4.9300(3) Å, *b* = 8.5641(3) Å, *c* = 5.0221(4) Å, *β* = 109.060(6)°					
Atom	Site	*x*	*y*	*z*	Occ_-refined_
Li	2c	0	0	0.5	0.958(8)
M	2c	0	0	0.5	0.042(8)
Li	2b	0	0.5	0	0.474(7)
M	2b	0	0.5	0	0.526(7)
Li	4g	0	0.163(4)	0	0.076(7)
M	4g	0	0.163(4)	0	0.924(8)
Li	4h	0	0.692(1)	0.5	0.970(4)
M	4h	0	0.692(1)	0.5	0.030(4)
O	4i	0.233(2)	0	0.216(2)	1
O	8j	0.252(3)	0.320(1)	0.228(1)	1

**Table:** 

(D-1)					
*S* = 2.20823, *R*_B_ = 5.329, *R*_F_ = 4.797, *R*_wp_ = 14.826					
*a* = 4.9560(2) Å, *b* = 8.5800(3) Å, *c* = 5.0154(1) Å, *β* = 109.140(2)°					
Atom	Site	*x*	*y*	*z*	Occ_-refined_
Li	2c	0	0	0.5	0.901(7)
M	2c	0	0	0.5	0.099(7)
Li	2b	0	0.5	0	0.013(9)
M	2b	0	0.5	0	0.997(9)
Li	4g	0	0.1667(6)	0	0.111(9)
M	4g	0	0.1667(6)	0	0.889(9)
Li	4h	0	0.676(3)	0.5	0.980(7)
M	4h	0	0.676(3)	0.5	0.020(7)
O	4i	0.237(3)	0	0.213(2)	1
O	8j	0.232(3)	0.341(1)	0.229(1)	1

**Table:** 

(D-2)					
*S* = 2.1854, *R*_B_ = 5.430, *R*_F_ = 4.695, *R*_wp_ = 15.224					
*a* = 4.9546(2) Å, *b* = 8.5868(2) Å, *c* = 5.0164(1) Å, *β* = 109.129(3)°					
Atom	Site	*x*	*y*	*z*	Occ_-refined_
Li	2c	0	0	0.5	0.912(6)
M	2c	0	0	0.5	0.088(6)
Li	2b	0	0.5	0	0.007(6)
M	2b	0	0.5	0	0.993(6)
Li	4g	0	0.1631(7)	0	0.092(6)
M	4g	0	0.1631(7)	0	0.908(6)
Li	4h	0	0.670(3)	0.5	0.993(3)
M	4h	0	0.670(3)	0.5	0.007(3)
O	4i	0.231(2)	0	0.215(3)	1
O	8j	0.240(2)	0.3313(9)	0.236(2)	1

**Table:** 

(E-1)					
*S* = 2.0025, *R*_B_ = 3.911, *R*_F_ = 3.000, *R*_wp_ = 13.149					
*a* = 4.9556(8) Å, *b* = 8.5618(9) Å, *c* = 5.0235(5) Å, *β* = 109.09(1)°					
Atom	Site	*x*	*y*	*z*	Occ_-refined_
Li	2c	0	0	0.5	0.965(4)
M	2c	0	0	0.5	0.035(4)
Li	2b	0	0.5	0	0.453(2)
M	2b	0	0.5	0	0.547(2)
Li	4g	0	0.1716(3)	0	0.049(6)
M	4g	0	0.1716(3)	0	0.951(6)
Li	4h	0	0.604(1)	0.5	0.986(6)
M	4h	0	0.604(1)	0.5	0.014(6)
O	4i	0.228(2)	0	0.215(1)	1
O	8j	0.245(2)	0.3265(8)	0.2271(9)	1

**Table:** 

(E-2)					
*S* = 2.2591, *R*_B_ = 5.122, *R*_F_ = 2.480, *R*_wp_ = 15.030					
*a* = 4.9488(1) Å, *b* = 8.56458(1) Å, *c* = 5.02165(6) Å, *β* = 109.0637(1)°					
Atom	Site	*x*	*y*	*z*	Occ_-refined_
Li	2c	0	0	0.5	0.941(3)
M	2c	0	0	0.5	0.059(3)
Li	2b	0	0.5	0	0.514(6)
M	2b	0	0.5	0	0.486(6)
Li	4g	0	0.1623(3)	0	0.075(6)
M	4g	0	0.1623(3)	0	0.925(6)
Li	4h	0	0.667(3)	0.5	0.998(2)
M	4h	0	0.667(3)	0.5	0.002(2)
O	4i	0.204(2)	0	0.219(1)	1
O	8j	0.257(2)	0.3142(9)	0.2274(8)	1

**Table:** 

(F-1)					
*S* = 1.8354, *R*_B_ = 3.655, *R*_F_ = 3.172, *R*_wp_ = 11.935					
*a* = 4.9386(5) Å, *b* = 8.5518(6) Å, *c* = 5.0174(3) Å, *β* = 108.997(7)°					
Atom	Site	*x*	*y*	*z*	Occ_-refined_
Li	2c	0	0	0.5	0.968(5)
M	2c	0	0	0.5	0.032(5)
Li	2b	0	0.5	0	0.509(2)
M	2b	0	0.5	0	0.491(2)
Li	4g	0	0.1687(6)	0	0.06(2)
M	4g	0	0.1687(6)	0	0.94(2)
Li	4h	0	0.664(3)	0.5	0.991(2)
M	4h	0	0.664(3)	0.5	0.009(2)
O	4i	0.272(2)	0	0.235(1)	1
O	8j	0.234(1)	0.3480(6)	0.2184(7)	1

**Table:** 

(F-2)					
*S* = 2.0422, *R*_B_ = 6.826, *R*_F_ = 4.087, *R*_wp_ = 15.235					
*a* = 4.93775(2) Å, *b* = 8.55114(3) Å, *c* = 5.01752(1) Å, *β* = 109.0085(4)°					
Atom	Site	*x*	*y*	*z*	Occ_-refined_
Li	2c	0	0	0.5	0.967(3)
M	2c	0	0	0.5	0.033(3)
Li	2b	0	0.5	0	0.541(5)
M	2b	0	0.5	0	0.459(5)
Li	4g	0	0.1687(8)	0	0.057(5)
M	4g	0	0.1687(8)	0	0.943(5)
Li	4h	0	0.664(2)	0.5	0.990(6)
M	4h	0	0.664(2)	0.5	0.010(6)
O	4i	0.268(1)	0	0.235(1)	1
O	8j	0.231(1)	0.3509(5)	0.2188(6)	1

Summary of the occupancy and occupancy difference of Li^+^ ions at 2c, 2b, 4g and 4h sites. The numbers correspond to those in Fig. 5: 1–3, 11, 12, 14, and 15 (yellow), 4, 10, 13, and 16 (pink), and 5–9 and 17–21 (green)

**Table:** 

Sample no.	Occupancy of Li^+^ ions calculated from Rietveld refinement analysis											
	Prepared by quenched cooling (A)				Prepared by slow cooling (B)				Ideal values for *C*2/*m* (C)			
	2c	2b	4g	4h	2c	2b	4g	4h	2c	2b	4g	4h
1	0.975	0.748	0.040	0.996	0.954	0.546	0.054	0.998	1	0.8	0	1
2	0.987	0.633	0.044	0.970	0.953	0.552	0.054	0.983	1	0.7	0	1
3	0.982	0.605	0.031	0.978	0.946	0.513	0.059	0.970	1	0.6	0	1
4	0.964	0.456	0.083	0.976	0.941	0.428	0.059	0.957	1	0.5	0	1
5	0.950	0.349	0.098	0.973	0.958	0.474	0.076	0.970	1	0.4	0	1
6	0.869	0.251	0.070	0.972	0.899	0.260	0.071	0.995	1	0.3	0	1
7	0.829	0.283	0.096	0.991	0.885	0.188	0.065	0.995	1	0.2	0	1
8	0.902	0.250	0.092	0.973	0.938	0.139	0.076	0.994	1	0.1	0	1
9	0.901	0.013	0.111	0.980	0.912	0.007	0.092	0.993	1	0	0	1
10	0.965	0.464	0.049	0.986	0.957	0.519	0.070	0.997	1	0.6	0	1
11	0.991	0.516	0.090	0.983	0.980	0.485	0.109	0.977	1	0.6	0	1
12	0.982	0.498	0.040	0.986	0.979	0.464	0.083	0.985	1	0.6	0	1
13	0.968	0.509	0.052	0.999	0.957	0.511	0.057	0.970	1	0.6	0	1
14	0.961	0.703	0.007	0.986	0.930	0.597	0.047	0.975	1	0.8	0	1
15	0.970	0.639	0.037	0.984	0.949	0.568	0.044	0.972	1	0.7	0	1
16	0.949	0.481	0.068	0.981	0.952	0.403	0.076	0.958	1	0.5	0	1
17	0.951	0.348	0.073	0.972	0.962	0.361	0.052	0.987	1	0.4	0	1
18	0.937	0.258	0.065	0.966	0.950	0.296	0.050	0.981	1	0.3	0	1
19	0.877	0.169	0.087	0.975	0.939	0.181	0.062	0.985	1	0.2	0	1
20	0.892	0.227	0.113	0.977	0.948	0.124	0.064	0.970	1	0.1	0	1
21	0.888	0.162	0.022	0.972	0.921	0.074	0.049	0.973	1	0	0	1

**Table:** 

Sample no.	Occupancy difference of Li^+^ ions							
	Occupancy difference at 2c site		Occupancy difference at 2b site		Occupancy difference at 4g site		Occupancy difference at 4h site	
	(D) = (A) − (C)	(E) = (B) − (C)	(F) = (A) − (C)	(G) = (B) − (C)	(H) = (A) − (C)	(I) = (B) − (C)	(J) = (A) − (C)	(K) = (B) − (C)
1	0.03	0.05	0.05	0.25	−0.04	−0.05	0.00	−0.05
2	0.01	0.05	0.07	0.15	−0.04	−0.05	0.03	−0.01
3	0.02	0.05	−0.01	0.09	−0.03	−0.06	0.02	−0.12
4	0.04	0.06	0.04	0.07	−0.08	−0.06	0.02	−0.02
5	0.05	0.04	0.05	−0.07	−0.10	−0.05	0.03	−0.09
6	0.13	0.10	0.05	0.04	−0.07	−0.07	0.03	−0.01
7	0.17	0.12	−0.08	0.01	−0.10	−0.07	0.01	−0.01
8	0.10	0.06	−0.15	−0.04	−0.09	−0.08	0.03	−0.03
9	0.10	0.09	−0.01	−0.01	−0.11	−0.09	0.02	−0.01
10	0.04	0.04	0.14	0.08	−0.05	−0.07	0.01	0.00
11	0.01	0.02	0.08	0.12	−0.09	−0.11	0.02	−0.02
12	0.02	0.02	0.10	0.14	−0.04	−0.08	0.01	−0.05
13	0.03	0.04	0.09	0.09	−0.05	−0.06	0.00	−0.03
14	0.04	0.07	0.10	0.20	−0.01	−0.05	0.01	−0.02
15	0.03	0.05	0.06	0.13	−0.04	−0.04	0.02	−0.05
16	0.05	0.05	0.02	0.10	−0.07	−0.08	0.02	−0.02
17	0.05	0.04	0.05	0.04	−0.07	−0.05	0.03	0.00
18	0.07	0.05	0.04	0.00	−0.07	−0.05	0.03	0.00
19	0.12	0.06	0.03	0.02	−0.09	−0.06	0.03	−0.02
20	0.11	0.05	−0.13	−0.02	−0.11	−0.06	0.02	−0.08
21	0.11	0.08	−0.16	0.07	−0.02	−0.05	0.03	−0.07

**Fig. 9:**
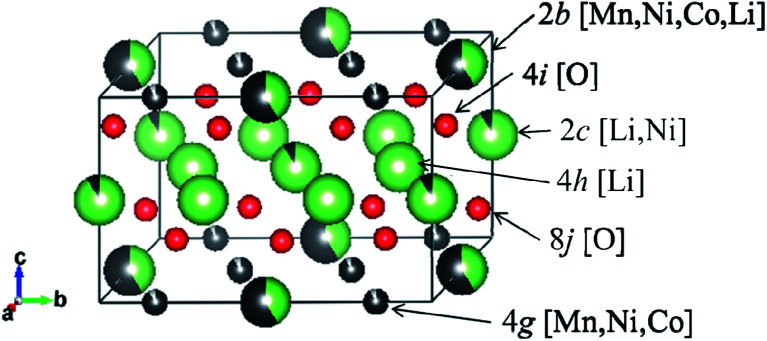
Definition of the sites occupied by Li^+^ ions in the space group of *C*2/*m*.

The occupancies of Li^+^ ions in the 2c, 2b, 4g and 4h sites, whose definitions are explained in Fig. 9 and which were analyzed with Rietveld refinement analysis, of the samples prepared by quenching and slow cooling are summarized in Table 4. In order to identify the difference in the crystal structure of the samples prepared by different cooling processes, the occupancies of the Li^+^ ions in the 2c, 2b, 4g and 4h sites of the samples were compared with the ideal values for the *C*2/*m* space group in each composition, and the values are listed in Table 4. By comparing the occupancies listed in Table 4, we tried to find the trend of the preferable cooling process to observe high discharge capacity. Unfortunately, no trend could be found by comparing the occupancies at each site. So, we considered the comparison of the total values of difference between the experimental and ideal values at the 2c, 2b, 4g and 4h sites in each sample to quantify the degree of crystallinity of the samples for the *C*2/*m* space group. The total difference is defined by eqn (2). The definitions of D, E, F, G, H, I, J and K are the same as those in Table 4.

2 Total difference = |D| + |F| + |H| + |J| for the samples prepared by quenching = |E| + |G| + |I| + |K| for the samples prepared by slow cooling

**Fig. 10:**
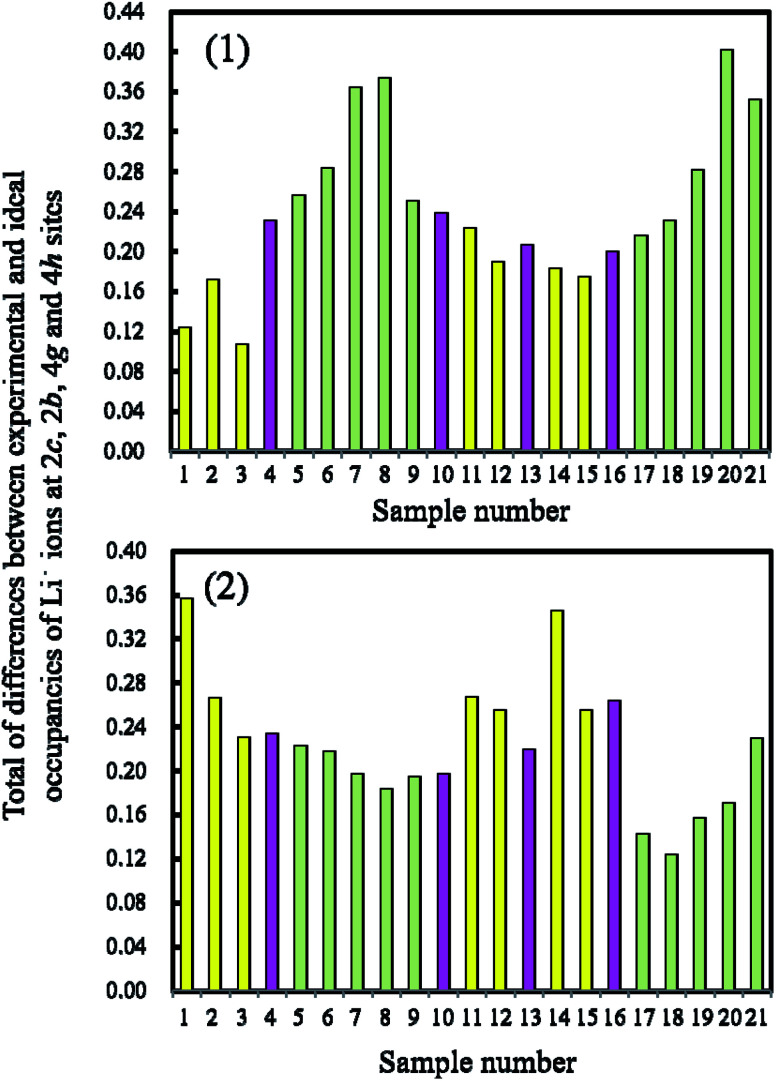
The total differences between the experimental and theoretical occupancies of Li^+^ ions at 2c, 2b, 4g and 4h sites in the LLO samples. Experimental values were obtained with the samples prepared with the (1) quenched cooling and (2) slow cooling processes. Yellow, pink and green colors correspond to those in Fig. 5, respectively. The experimental and theoretical occupancies of Li^+^ ions at the 2c, 2b, 4g and 4h sites are taken from Table 3.

The total differences in the samples prepared by the quenching and slow cooling processes were plotted. Fig. 10 shows these corresponding plots for the LLO samples prepared by (A) quenching and (B) slow cooling processes. The definition of the colors for the bars is the same as that used for the difference in the discharge capacity between the samples prepared by quenching and slow cooling in Fig. 5. In the figure, lower bars mean that the crystal structure of the LLO samples take a more ideal structure with the *C*2/*m* space group. Conversely, when the samples showed higher bars, the samples take on a more disordered structure for the *C*2/*m* space group. In (A), samples 1, 2, 3, 11, 12, 14 and 15 (yellow bars) exhibiting higher discharge capacity with the quenched cooling process showed a lower total difference of occupancies among the samples. On the other hand, samples 5, 6, 7, 8, 9, 17, 18, 19, 20 and 21 (green bars) showing higher discharge capacity (when the slow cooling process was used) showed a higher total difference of occupancies among the samples. In (B), samples 1, 2, 3, 11, 12, 14 and 15 (yellow bars) have a higher total difference, and samples 5, 6, 7, 8, 9, 17, 18, 19, 20 and 21 (green bars) have a lower total difference. Samples 1, 2, 3, 11, 12, 14 and 15 tend to approach the ideal structure with the *C*2/*m* space group when the samples are quenched. Conversely, when the samples are cooled slowly, their structures develop a low degree of ordering of the crystal structure with the *C*2/*m* space group. Samples 5, 6, 7, 8, 9, 17, 18, 19, 20 and 21 show the opposite trend. In addition, as the LLO samples have a more ideal structure with the *C*2/*m* space group, the samples show higher discharge capacity. Samples 1, 2, 3, 11, 12, 14 and 15 contain a Li_2_MnO_3_ content of over 60%. The content rate of Li_2_MnO_3_ has control over the degree of crystallinity, and the better cooling process for obtaining higher capacity. It has been observed that the LLOs containing the Li_2_MnO_3_ content of over 60% have the crystal structure, which has the ideal form close to the *C*2/*m* space group, in the quenched cooling process. However, the LLOs containing the Li_2_MnO_3_ at less 60% can also have this crystal structure after the slow cooling process. The reason why this is the case is not understood. This question will be analyzed with molecular dynamics and density functional theory in our next step.

## 4. Conclusions

In this study, in order to clarify the effect of the cooling process of Li-rich solid-solution layered oxide (LLO) materials on the cathode performance in lithium ion batteries, two types of cooling processes were compared. These two processes were (1) the quenching of the LLO samples with liquid nitrogen, and (2) the slow cooling with the controlled decreasing rate of the temperature in the furnace where the LLO samples were placed. The two cooling processes were each applied to the LLO samples, and then the charging/discharging cycle performance of the LLO samples was obtained. From these results, the necessity of the quenching process for the LLO samples to exhibit high charging/discharging capacities depended on the composition of the LLO samples. As the composition of the tested LLO samples deviated from the composition of the Li[Li_0.20_Mn_0.58_Ni_0.18_Co_0.04_]O_2_ (Li_2_MnO_3_ (60%)–LiNi_0.5_Mn_0.5_O_2_ (30%)–LiNi_1/3_Co_1/3_Mn_1/3_O_2_ (10%)) sample, which exhibited the highest charging/discharging capacities among the tested LLO samples, the effect of the quenching process on the charging/discharging capacities became weaker. The LLO samples prepared with the slow cooling process exhibited higher charging/discharging capacities when compared with the LLO samples prepared with the quenching process. The structural analysis of the pXRD patterns of the LLO samples indicated that the samples exhibiting high charging/discharging capacities had a high degree of crystallinity of LLO samples for the space group *C*2/*m*. This means that there was a low degree of cation mixing between the Li^+^ and Ni^2+^ ions, regardless of the type of cooling process. This study showed that the tendency that a high degree of crystallinity of LLO samples for the space group *C*2/*m* was needed for the LLO samples to exhibit high charging/discharging capacities. Furthermore, it was found that the cooling process is not restricted for the LLO samples to exhibit high capacities to the quenching process, and which process is better depended on the composition of the LLO samples. Even in the cooling process for the LLO samples that had the same percentage of Li_2_MnO_3_ in the LLO samples, the necessity of the quenching process was different. There were also samples that needed the slow cooling process to exhibit higher capacities. The reason why the cooling process required for high capacities is different for samples, depending on the composition, is not clear. The elucidation of this question is in progress.

## Conflicts of interest

There are no conflicts to declare.

## Supplementary Material

RA-011-D0RA06680J-s001
