# Supplementary material for: The effect of cooling process on the structure and charge/discharge capacities of Li-rich solid-solution layered oxide cathode materials for the Li-ion battery
Source: RSC Adv. 2021 Jan 6;11(3):1715–28. doi: 10.1039/d0ra06680j (PMC8693521; doi:10.1039/d0ra06680j)
Supplement: RA-011-D0RA06680J-s001 [file RA-011-D0RA06680J-s001.pdf]

Electronic Supplementary Material (ESI) for RSC Advances.
This journal is © The Royal Society of Chemistry 2020

Supporting Information

Effect of the Cooling Process on the Structure and Charge/Discharge Capacities of Li-rich Solid-Solution Layered Oxide Cathode Materials for Li-Ion Battery

Fumihiro Nomura [a], Tatsuya Watanabe [a], Hiroya Ochiai [a], Takao Gunji [a, b], Takeshi Hagiwara [b], Jianfei Wu [c], Futoshi Matsumoto [a, b] *

[a] Department of Materials and Life Chemistry, Kanagawa University, 3-27-1, Rokkakubashi, Kanagawa-ku, Yokohama, Kanagawa 221-8686, Japan

[b] Research Institute for Engineering, Kanagawa University, 3-27-1, Rokkakubashi, Kanagawa-ku, Yokohama, Kanagawa 221-8686, Japan

[c] Qingdao Industrial Energy Storage Research Institute, Qingdao Institute of Bioenergy and Bioprocess Technology, Chinese Academy of Sciences, No. 189 Songling Road, 266101 Qingdao, China

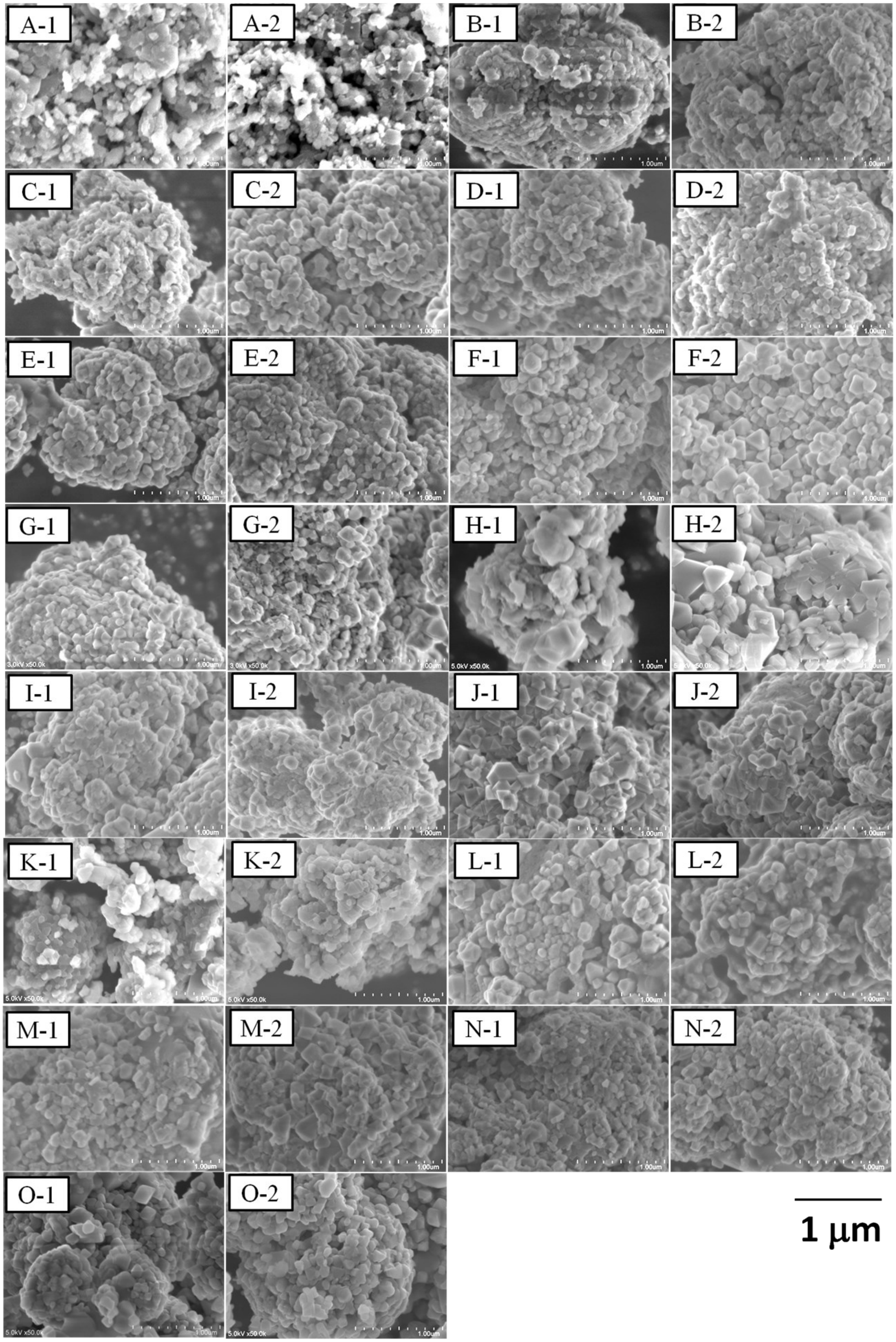
A-1
A-2
B-1
B-2
C-1
C-2
D-1
D-2
E-1
E-2
F-1
F-2
G-1
G-2
H-1
H-2
I-1
I-2
J-1
J-2
K-1
K-2
L-1
L-2
M-1
M-2
N-1
N-2
O-1
O-2
1 μm

Fig. S1 SEM images of the LLO samples prepared by (A-1, B-1, C-1, D-1, E-1, F-1, G-1, H-1, I-1, J-1, K-1, L-1, M-1, N-1 and O-1) quenched cooling with liquid nitrogen and (A-2, B-2, C-2, D-2, E-2, F-2, G-2, H-2, I-2, J-2, K-2, L-2, M-2, N-2 and O-2) slow cooling of the calcined material at a controlled rate of 25 °C h^{-1} in the furnace from 900 to 25℃. (A) $Li_{1.23}Ni_{0.14}Mn_{0.61}Co_{0.02}O_2$ (sample 2), (B) $Li_{1.17}Ni_{0.22}Mn_{0.56}Co_{0.05}O_2$ (sample 4), (C) $Li_{1.1}Ni_{0.31}Mn_{0.51}Co_{0.08}O_2$ (sample 6), (D) $Li_{1.07}Ni_{0.35}Mn_{0.48}Co_{0.1}O_2$ (sample 7), (E) $Li_{1.03}Ni_{0.39}Mn_{0.46}Co_{0.12}O_2$ (sample 8), (F) $Li_{1.2}Ni_{0.16}Mn_{0.57}Co_{0.07}O_2$ (sample 11), (G) $Li_{1.2}Ni_{0.15}Mn_{0.55}Co_{0.1}O_2$ (sample 12), (H) $Li_{1.27}Ni_{0.1}Mn_{0.63}O_2$ (sample 14), (I) $Li_{1.23}Ni_{0.14}Mn_{0.61}Co_{0.02}O_2$ (sample 15), (J) $Li_{1.17}Ni_{0.22}Mn_{0.56}Co_{0.05}O_2$ (sample 16), (K) $Li_{1.2}Ni_{0.27}Mn_{0.53}Co_{0.07}O_2$ (sample 17), (L) $Li_{1.2}Ni_{0,31}Mn_{0.51}Co_{0.08}O_2$ (sample 18), (M) $Li_{1.07}Ni_{0.35}Mn_{0.48}Co_{0.1}O_2$ (sample 19), (N) $Li_{1.03}Ni_{0.39}Mn_{0.46}Co_{0.12}O_2$ (sample 20) and (O) $Li_1Ni_{0.43}Mn_{0.43}Co_{0.14}O_2$ (sample 21).

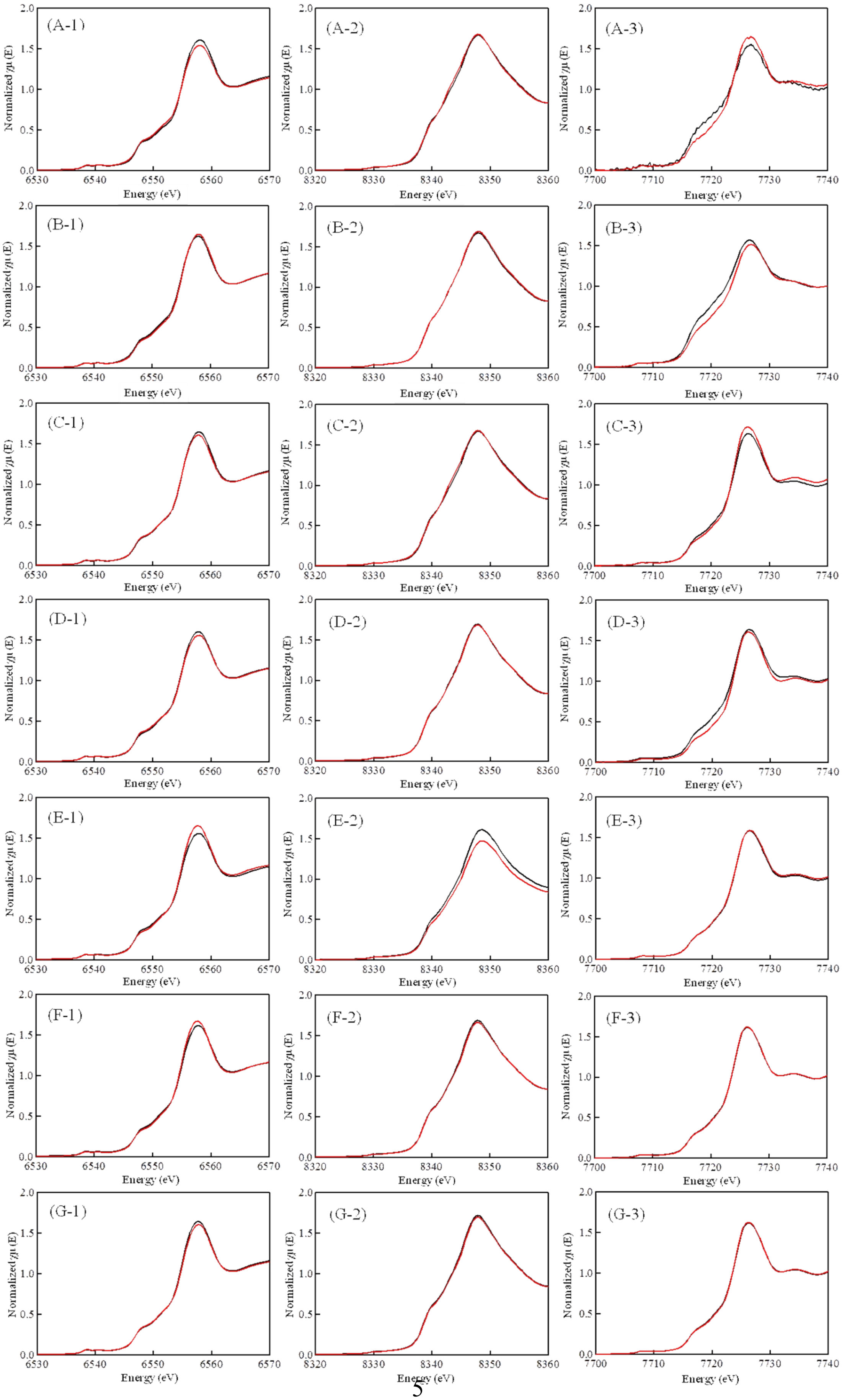

(A-1)
(A-2)
(A-3)
(B-1)
(B-2)
(B-3)
(C-1)
(C-2)
(C-3)
(D-1)
(D-2)
(D-3)
(E-1)
(E-2)
(E-3)
(F-1)
(F-2)
(F-3)
(G-1)
(G-2)
(G-3)
Normalized χμ(E)
Energy (eV)

Fig. S2 K-edge XANES spectra of (A-1, ···, G-1) Mn, (A-2, ···, G-2) Ni and (A-3, ···, G-3) Co ions of the samples 2 (A), 3 (B), 4 (C), 5 (D), 6 (E), 7 (F) and 8 (G) prepared by the quenched cooling with liquid nitrogen (black line) and the slow cooling in the furnace at a controlled cooling rate of 25 °C h^{-1}(red line).

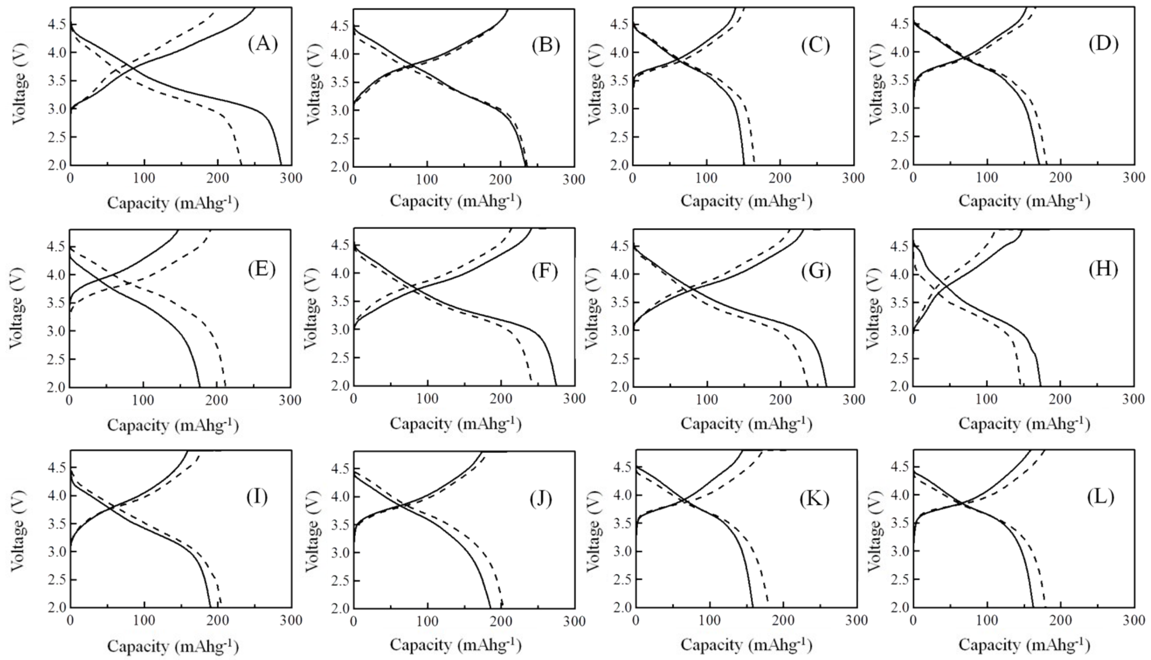

Fig. S3 Charging/discharging voltage-capacity curves obtained at the 10th cycle with samples 2 (A), 4 (B), 6 (C), 7 (D), 8 (E), 11 (F), 12 (G), 15 (H), 16 (I), 18 (J), 19 (K), and 20 (L) prepared by the quenched cooling with liquid nitrogen (solid lines) and the slow cooling in the furnace at a controlled rate of 25 °C h^{-1} (dotted lines). The charging/discharging rate was 0.1 C.

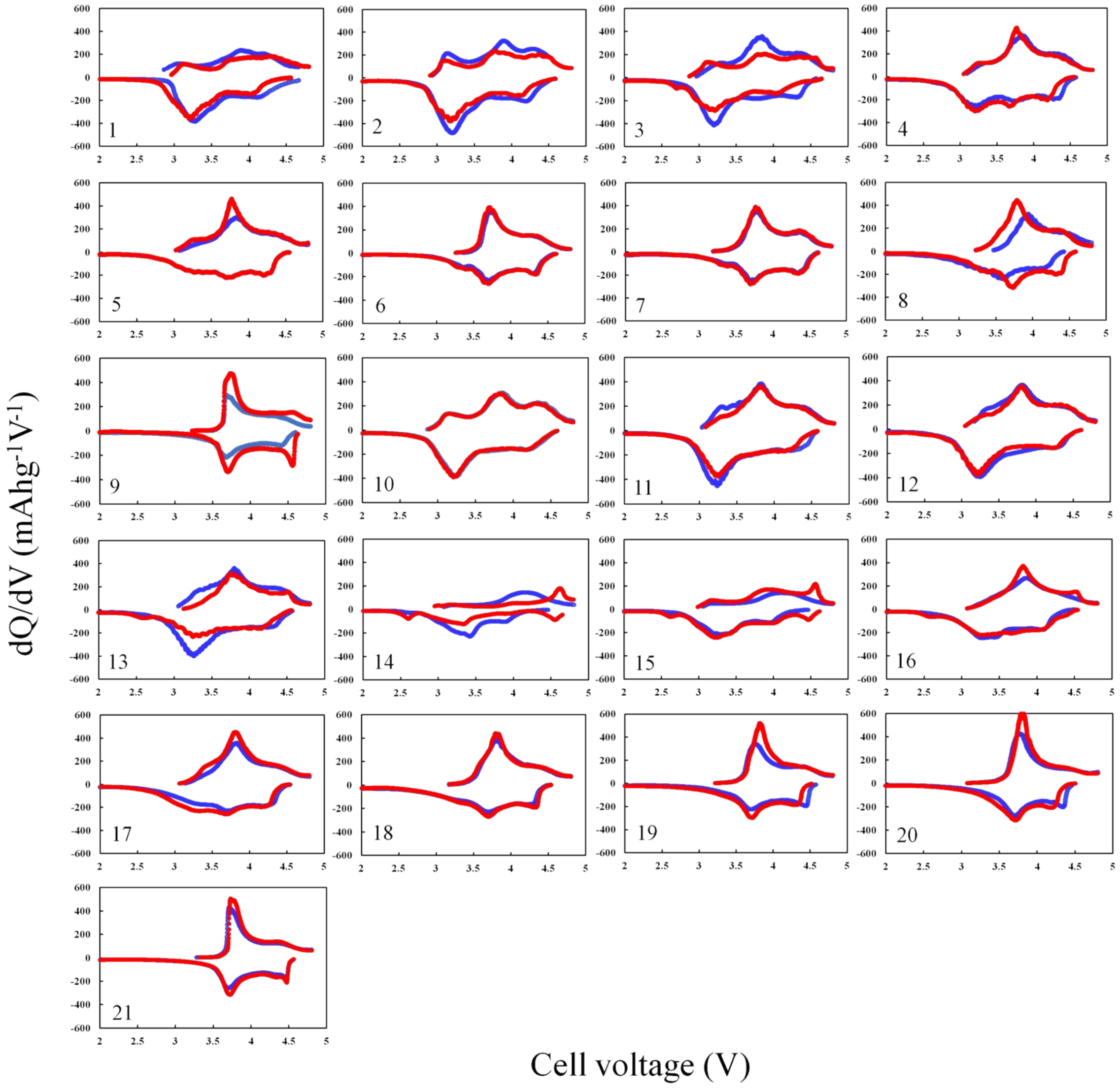

Fig. S4 dQ/dV *vs.* cell voltage curves obtained from charging/discharging curves at the 10th cycle. Blue and red curves correspond to the samples prepared by the quenched cooling and slow cooling processes, respectively. The number in each figure indicates the corresponding sample number in Table 1

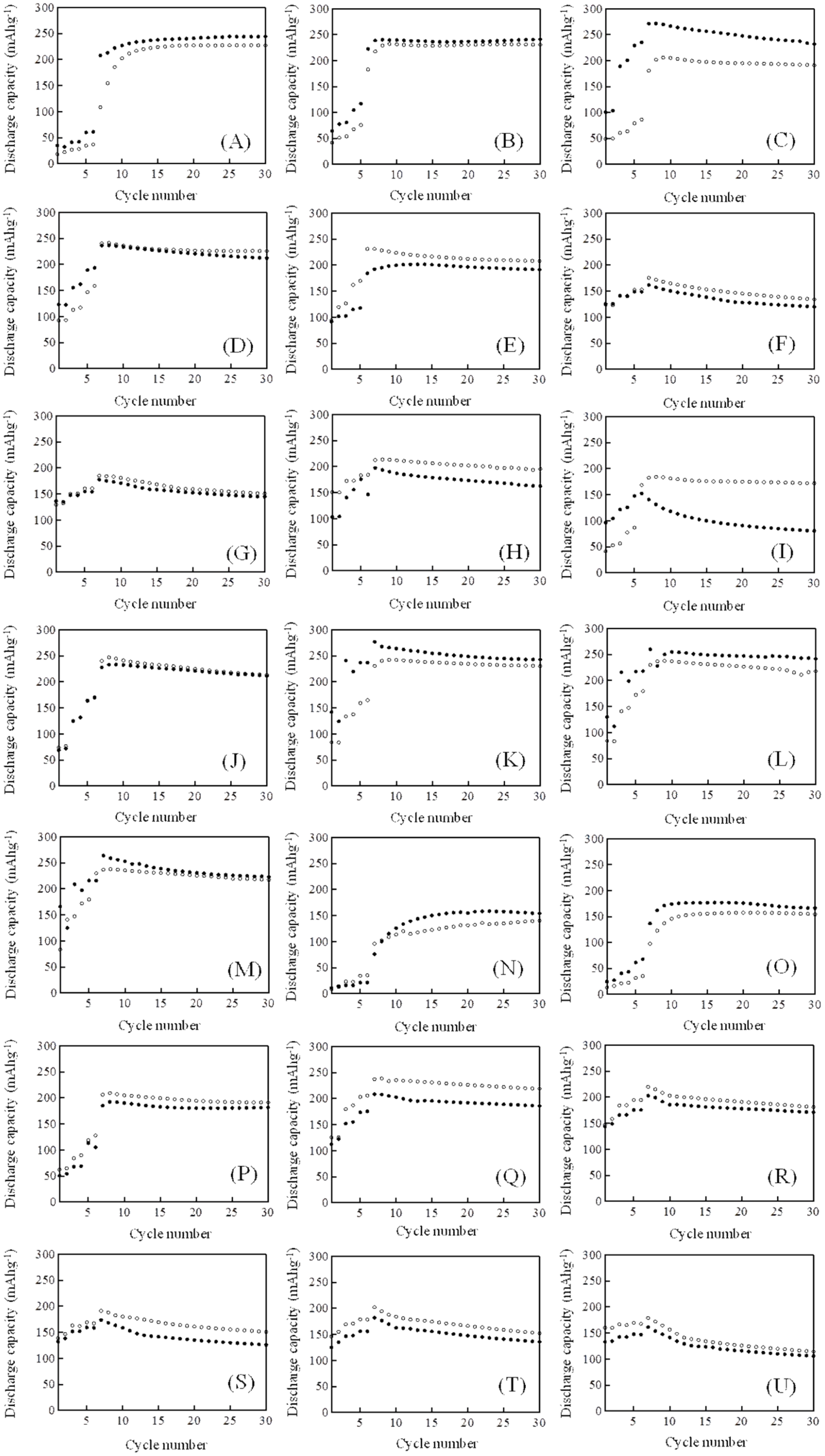
Discharge capacity (mAhg⁻¹)
Cycle number
(A)
(B)
(C)
(D)
(E)
(F)
(G)
(H)
(I)
(J)
(K)
(L)
(M)
(N)
(O)
(P)
(Q)
(R)
(S)
(T)
(U)

Fig. S5 Charging/discharging cycle performance obtained at a charging/discharging current density of 0.1 C-rate for the LLO samples prepared by (●) the quenched cooling with liquid nitrogen and (○) the slow cooling in the furnace at a controlled rate of 25 °C h^{-1}. Samples: 1 (A), 2 (B), 3 (C), 4 (D), 5 (E), 6 (F), 7 (G), 8 (H), 9 (I), 10 (J), 11 (K), 12 (L), 13 (M), 14 (N), 15 (O), 16 (P), 17 (Q), 18 (R), 19 (S), 20 (T) and 21 (U).

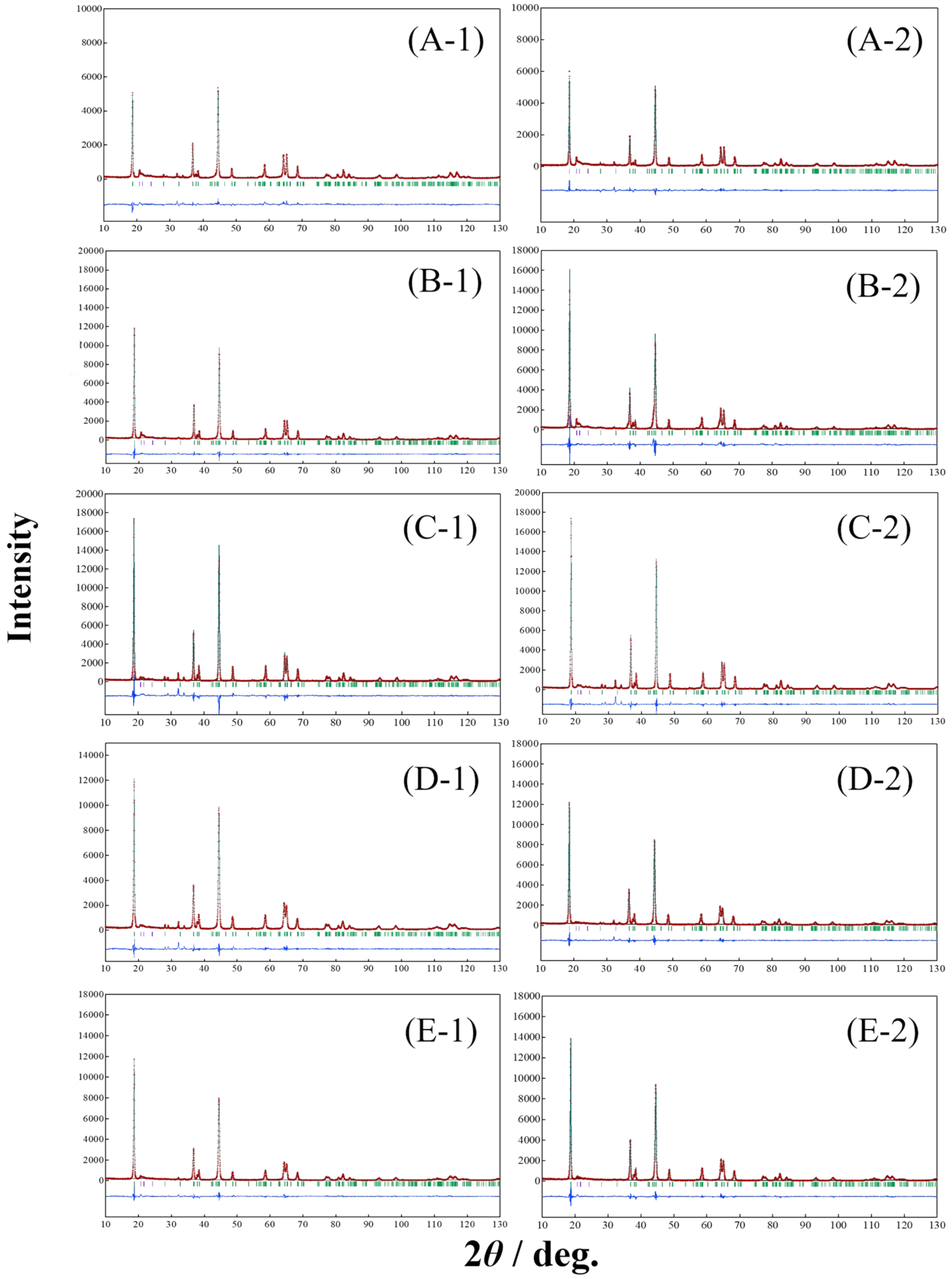
(A-1)
(A-2)
(B-1)
(B-2)
(C-1)
(C-2)
(D-1)
(D-2)
(E-1)
(E-2)
Intensity
2θ / deg.

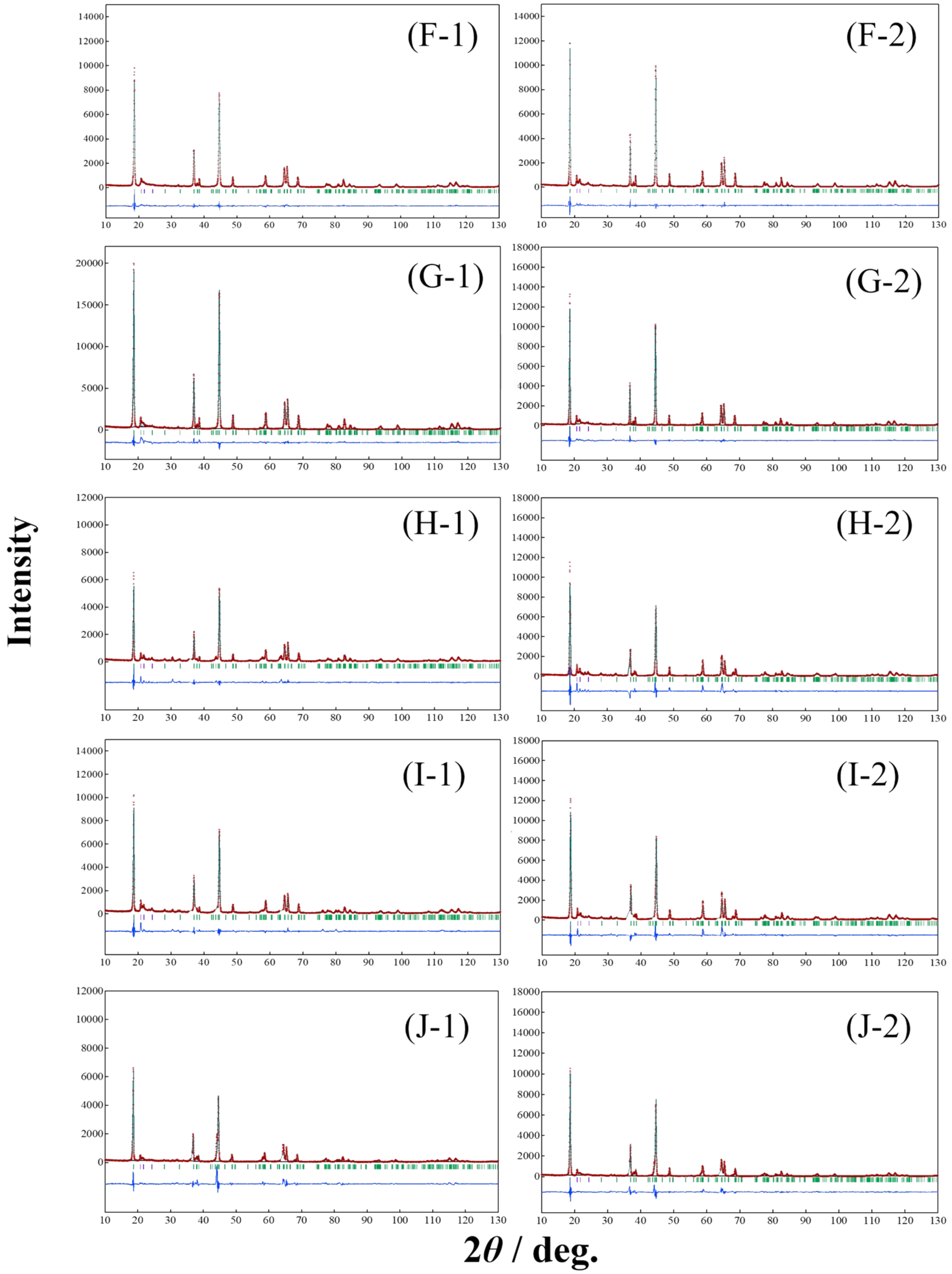

(F-1)
(F-2)
(G-1)
(G-2)
(H-1)
(H-2)
(I-1)
(I-2)
(J-1)
(J-2)
Intensity
2θ / deg.

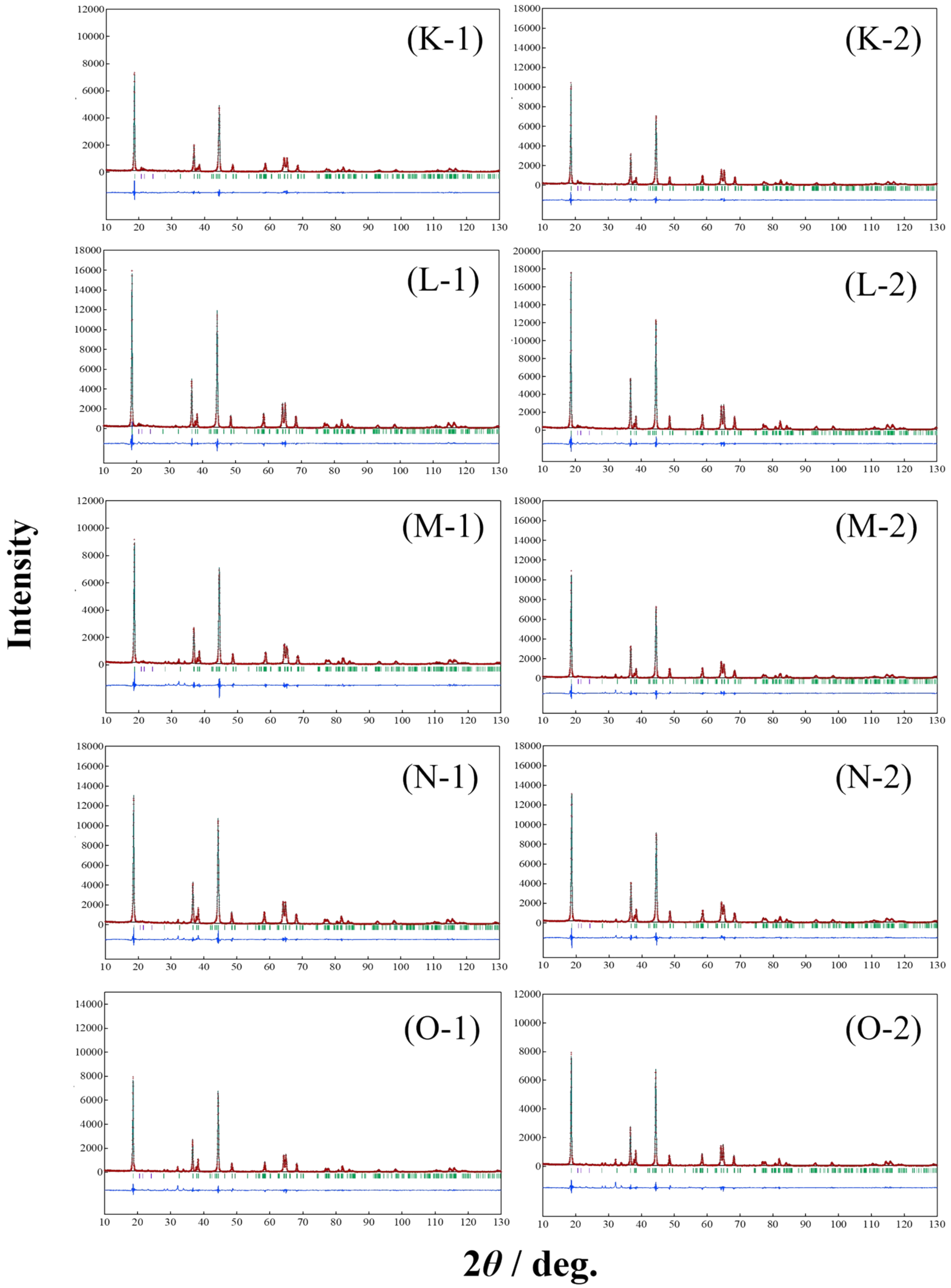

Fig. S6 XRD Rietveld refinement profiles of samples prepared by (1) the quenched cooling with liquid nitrogen and (2) slow cooling in the furnace at a controlled rate of 25

°C h^{-1}. ( ) observed, ( ) calculated, ( ) the residual difference of both. The green vertical marks indicate the position of the Bragg reflections. * is the peaks approximately 20-25° that result from ordering of Li^+ ions in the transition metal layers. Samples 2 (A), 4 (B), 6 (C), 7 (D), 8 (E), 11 (F), 12 (G), 14 (H), 15 (I), 16 (J), 17 (K), 18 (L), 19 (M), 20 (N) and 21 (O).

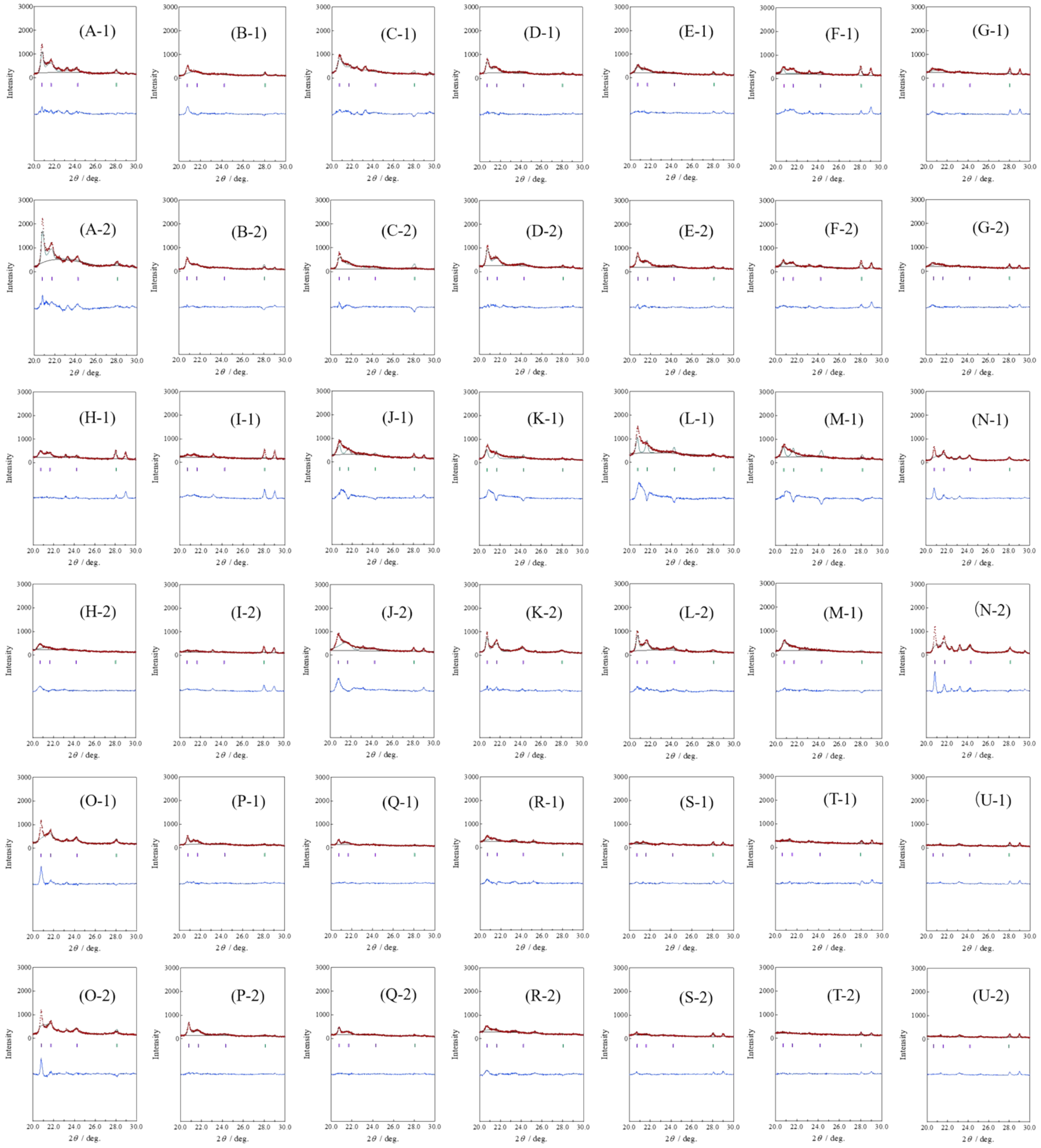

Fig. S7 XRD Rietveld refinement profiles from 20 to 30° of the LLO samples prepared by (1) the quenched cooling with liquid nitrogen and (2) the slow cooling in the furnace at a controlled rate of 25 °C h^{-1}. ( ) observed, ( ) calculated, ( ) the difference of both. The vertical marks indicate the position of the Bragg reflections. (A) sample 1, (B) sample 2, (C) sample 3, (D) sample 4, (E) sample 5, (F) sample 6, (G) sample 7, (H)

sample 8, (I) sample 9, (J) sample 10, (K) sample 11, (L) sample 12, (M) sample 13, (N) sample 14, (O) sample 15, (P) sample 16, (Q) sample 17, (R) sample 18, (S) sample 19,(T) sample 20 and (U) sample 21.

Table S1 Summary of the bulk and surface atomic ratios of Mn, Ni and Co in LLO samples. Nos. 1-21 were prepared with the quenched cooling and slow cooling processes, which were evaluated with XRF and XPS, respectively, and the difference in the surface and bulk atomic percentages ratios and the difference in atomic percentages of samples prepared by quenched cooling and slow cooling processes.

Sample No.	Bulk atomic ratios (A)						Surface atomic ratios (B)					
	Quenched sample			Slow cooled sample			Quenched sample			Slow cooled sample		
	Mn	Co	Ni	Mn	Co	Ni	Mn	Co	Ni	Mn	Co	Ni
1	0.86	0.00	0.14	0.86	0.00	0.14	0.53	0.00	0.47	0.40	0.00	0.60
2	0.79	0.02	0.19	0.79	0.02	0.19	0.52	0.03	0.45	0.42	0.01	0.56
3	0.74	0.04	0.22	0.73	0.04	0.23	0.41	0.18	0.42	0.31	0.19	0.50
4	0.67	0.06	0.27	0.67	0.06	0.27	0.44	0.05	0.51	0.35	0.14	0.52
5	0.62	0.08	0.30	0.62	0.08	0.31	0.30	0.11	0.59	0.22	0.13	0.65
6	0.56	0.09	0.34	0.56	0.09	0.34	0.38	0.08	0.54	0.30	0.08	0.63
7	0.51	0.11	0.38	0.51	0.11	0.38	0.40	0.06	0.55	0.31	0.09	0.60
8	0.46	0.13	0.41	0.47	0.13	0.40	0.38	0.08	0.54	0.38	0.07	0.55
9	0.40	0.20	0.41	0.39	0.20	0.40	0.32	0.07	0.61	0.32	0.08	0.61
10	0.77	0.00	0.23	0.76	0.00	0.24	0.52	0.00	0.48	0.52	0.00	0.48
11	0.71	0.09	0.21	0.71	0.09	0.20	0.43	0.09	0.48	0.42	0.07	0.51
12	0.68	0.13	0.19	0.70	0.12	0.19	0.40	0.10	0.50	0.43	0.06	0.51
13	0.66	0.17	0.17	0.67	0.17	0.16	0.41	0.16	0.43	0.47	0.18	0.36
14	0.82	0.09	0.09	0.81	0.10	0.09	0.49	0.14	0.37	0.37	0.17	0.46
15	0.74	0.11	0.14	0.73	0.12	0.15	0.68	0.09	0.23	0.56	0.15	0.30
16	0.64	0.14	0.22	0.63	0.14	0.23	0.40	0.17	0.43	0.36	0.14	0.49
17	0.57	0.15	0.27	0.58	0.15	0.27	0.46	0.19	0.35	0.37	0.11	0.51
18	0.52	0.17	0.31	0.53	0.16	0.31	0.32	0.14	0.54	0.31	0.13	0.56
19	0.47	0.19	0.34	0.47	0.19	0.34	0.37	0.15	0.48	0.36	0.12	0.52
20	0.44	0.19	0.37	0.44	0.20	0.37	0.29	0.14	0.58	0.30	0.13	0.56
21	0.39	0.20	0.41	0.39	0.20	0.40	0.31	0.13	0.55	0.32	0.12	0.55

Sample No.	Difference in surface and bulk atomic percentages (%) [a]						Difference in surface and bulk atomic percentages of samples prepared by quenched and slow cooling processes (%) [b]		
	Quenched samples (C)			Slow cooled sample (D)					
	Mn	Co	Ni	Mn	Co	Ni	Mn	Co	Ni
1	-38	0	236	-53	0	329	15	0	-93
2	-34	50	136	-47	-50	195	13	0	-59
3	-45	350	91	-58	375	117	13	-25	-26
4	-34	-17	89	-48	133	93	14	-150	-2
5	-52	27	97	-65	63	110	13	-36	-13
6	-32	-11	59	-46	-11	85	14	0	-26
7	-22	-45	45	-39	-18	58	17	-27	-13
8	-17	-38	32	-19	-46	38	2	8	-6
9	-20	-65	49	-18	-60	53	-2	-5	-4
10	-32	0	109	-32	0	100	0	0	9
11	-39	0	129	-41	-22	155	2	-2	-26
12	-41	-23	163	-39	-50	168	-9	27	-5
13	-38	-6	153	-30	6	125	-8	-12	28
14	-40	56	311	-54	70	411	14	-14	-100
15	-8	-18	64	-23	25	100	15	-43	-36
16	-38	21	95	-43	0	113	5	21	-18
17	-19	27	30	-36	-27	89	17	54	-59
18	-38	-15	74	-42	-19	81	4	4	-7
19	-21	-21	41	-23	-37	53	3	16	-12
20	-34	-26	57	-32	-35	51	-2	9	6
21	-21	-35	34	-18	-40	38	-2	5	-4

a) Calculated as {(B) - (A)/(A)} x 100.

b) Calculated as (C) - (D).

Table S2 The refined structural parameters of LLO samples obtained from the Rietveld refinement with the space of *C*2/m. The LLO samples were prepared by (1) the quenched cooling with liquid nitrogen and (2) the slow cooling in the furnace at a controlled rate of 25 °C h^{-1}. Samples: 2 (A), 4 (B), 6 (C), 7 (D), 8 (E), 11 (F),12 (G), 14 (H), 15 (I), 16 (J), 17 (K), 18 (L), 19 (M), 20 (N) and 21 (O).

(A-1)

$S=1.9770$ $R_B=7.855$ $R_F=6.173$ $R_{wp}=15.152$

a=4.9445(4) Å, b=8.5614(5) Å, c=5.0377(2) Å, β=109.211(5) °

atom	site	x	y	z	$occ_{\text{-refined}}$
Li	2c	0	0	0.5	0.987(5)
M	2c	0	0	0.5	0.013(5)
Li	2b	0	0.5	0	0.633(5)
M	2b	0	0.5	0	0.367(5)
Li	4g	0	0.1663(2)	0	0.044(5)
M	4g	0	0.1663(2)	0	0.956(5)
Li	4h	0	0.675(2)	0.5	0.970(1)
M	4h	0	0.675(2)	0.5	0.030(1)
O	4i	0.234(1)	0	0.2273(7)	1
O	8j	0.254(1)	0.3209(5)	0.2395(7)	1

(A-2)

$S=1.7322$ $R_B=5.035$ $R_F=4.98$ $R_{wp}=15.147$

a=4.9420(4) Å, b=8.5628(5) Å, c=5.0305(2) Å, β=108.985(5) °

atom	site	x	y	z	$occ_{\text{-refined}}$
Li	2c	0	0	0.5	0.953(3)
M	2c	0	0	0.5	0.047(3)
Li	2b	0	0.5	0	0.673(2)
M	2b	0	0.5	0	0.327(2)
Li	4g	0	0.1663(2)	0	0.054(2)
M	4g	0	0.1663(2)	0	0.946(2)
Li	4h	0	0.675(2)	0.5	0.983(2)
M	4h	0	0.675(2)	0.5	0.017(2)
O	4i	0.237(2)	0	0.2192(8)	1
O	8j	0.265(3)	0.3212(5)	0.2306(8)	1

(B-1)

$S=1.6248$ $R_B=2.445$ $R_F=2.830$ $R_{wp}=10.854$

a=4.9499(2) Å, b=8.5784(3) Å, c=5.0253(1) Å, β=109.06(4) °

atom	site	x	y	z	$occ_{\text{-refined}}$
Li	2c	0	0	0.5	0.964(5)
M	2c	0	0	0.5	0.036(5)
Li	2b	0	0.5	0	0.456(5)
M	2b	0	0.5	0	0.543(5)
Li	4g	0	0.1647(2)	0	0.083(3)
M	4g	0	0.1627(2)	0	0.917(3)
Li	4h	0	0.684(1)	0.5	0.976(3)
M	4h	0	0.684(1)	0.5	0.024(3)
O	4i	0.219(2)	0	0.218(1)	1
O	8j	0.253(1)	0.3177(6)	0.2289(7)	1

(B-2)

$S=2.2912$ $R_B=4.063$ $R_F=4.279$ $R_{wp}=14.476$

a=4.93863(2) Å, b=8.55803(3) Å, c=5.02207(1) Å, β=109.012(4) °

atom	site	x	y	z	$occ_{\text{-refined}}$
Li	2c	0	0	0.5	0.964(5)
M	2c	0	0	0.5	0.036(5)
Li	2b	0	0.5	0	0.487(7)
M	2b	0	0.5	0	0.513(7)
Li	4g	0	0.1647(2)	0	0.059(7)
M	4g	0	0.1627(2)	0	0.941(7)
Li	4h	0	0.684(1)	0.5	0.965(3)
M	4h	0	0.684(1)	0.5	0.035(3)
O	4i	0.229(3)	0	0.208(1)	1
O	8j	0.245(2)	0.3178(8)	0.2299(8)	1

(C-1)

$S=2.7191$ $R_B=8.721$ $R_F=5.573$ $R_{wp}=16.498$

a=4.95876(2) Å, b=8.56992(3) Å, c=5.01799(1) Å, β=109.2371(3)°

atom	site	x	y	z	$occ_{\text{-refined}}$
Li	2c	0	0	0.5	0.888(7)
M	2c	0	0	0.5	0.112(7)
Li	2b	0	0.5	0	0.284(7)
M	2b	0	0.5	0	0.7158(7)
Li	4g	0	0.1657(3)	0	0.088(7)
M	4g	0	0.1657(3)	0	0.912(7)
Li	4h	0	0.657(2)	0.5	0.975(5)
M	4h	0	0.657(2)	0.5	0.025(5)
O	4i	0.230(3)	0	0.228(1)	1
O	8j	0.244(2)	0.3184(8)	0.2230(9)	1

(C-2)

$S=2.2164$ $R_B=4.211$ $R_F=4.576$ $R_{wp}=14.085$

a=4.9583(2) Å, b=8.5789(3) Å, c=5.0182(1) Å, β=109.241(3)°

atom	site	x	y	z	$occ_{\text{-refined}}$
Li	2c	0	0	0.5	0.899(7)
M	2c	0	0	0.5	0.101(7)
Li	2b	0	0.5	0	0.260(7)
M	2b	0	0.5	0	0.740(7)
Li	4g	0	0.1657(3)	0	0.071(7)
M	4g	0	0.1657(3)	0	0.929(7)
Li	4h	0	0.657(2)	0.5	0.995(4)
M	4h	0	0.657(2)	0.5	0.005(4)
O	4i	0.233(2)	0	0.230(6)	1
O	8j	0.245(2)	0.3190(5)	0.2242(8)	1

(D-1)

$S=1.9684$ $R_B=30123$ $R_F=3.692$ $R_{wp}=12.268$

a=4.9691(3) Å, b=8.5829(2) Å, c=5.0275(2) Å, β=109.226(4)°

atom	site	x	y	z	$occ_{\text{-refined}}$
Li	2c	0	0	0.5	0.829(4)
M	2c	0	0	0.5	0.171(4)
Li	2b	0	0.5	0	0.283(5)
M	2b	0	0.5	0	0.717(5)
Li	4g	0	0.1639(4)	0	0.096(5)
M	4g	0	0.1639(4)	0	0.904(5)
Li	4h	0	0.643(1)	0.5	0.991(5)
M	4h	0	0.643(1)	0.5	0.009(5)
O	4i	0.218(2)	0	0.224(1)	1
O	8j	0.242(2)	0.321(1)	0.228(1)	1

(D-2)

$S=1.7967$ $R_B=3.543$ $R_F=3.735$ $R_{wp}=11.941$

a=4.966(3) Å, b=8.5808(2) Å, c=5.0265(2) Å, β=109.223(4)°

atom	site	x	y	z	$occ_{\text{-refined}}$
Li	2c	0	0	0.5	0.871(1)
M	2c	0	0	0.5	0.129(1)
Li	2b	0	0.5	0	0.161(5)
M	2b	0	0.5	0	0.839(5)
Li	4g	0	0.1642(4)	0	0.064(5)
M	4g	0	0.1642(4)	0	0.936(5)
Li	4h	0	0.658(3)	0.5	0.994(5)
M	4h	0	0.658(3)	0.5	0.006(5)
O	4i	0.226(3)	0	0.228(2)	1
O	8j	0.248(2)	0.324(1)	0.224(1)	1

(E-1)

$S=2.1748$ $R_B=5.714$ $R_F=4.502$ $R_{wp}=14.475$

a=4.9584(2) Å, b=8.5706(4) Å, c=5.0179(2) Å, β=109.234(5)°

atom	site	x	y	z	$occ_{\text{-refined}}$
Li	2c	0	0	0.5	0.902(6)
M	2c	0	0	0.5	0.098(6)
Li	2b	0	0.5	0	0.250(6)
M	2b	0	0.5	0	0.750(6)
Li	4g	0	0.1639(4)	0	0.092(6)
M	4g	0	0.1639(4)	0	0.908(6)
Li	4h	0	0.673(1)	0.5	0.973(4)
M	4h	0	0.673(1)	0.5	0.023(4)
O	4i	0.224(3)	0	0.217(1)	1
O	8j	0.254(2)	0.324(1)	0.2271(8)	1

(E-2)

$S=1.6814$ $R_B=2.781$ $R_F=3.102$ $R_{wp}=10.835$

a=4.9581(3) Å, b=8.5811(4) Å, c=5.0269(2) Å, β=109.288(4)°

atom	site	x	y	z	$occ_{\text{-refined}}$
Li	2c	0	0	0.5	0.895(7)
M	2c	0	0	0.5	0.105(7)
Li	2b	0	0.5	0	0.156(6)
M	2b	0	0.5	0	0.844(6)
Li	4g	0	0.1640(3)	0	0.058(6)
M	4g	0	0.1640(3)	0	0.942(6)
Li	4h	0	0.681(2)	0.5	0.991(4)
M	4h	0	0.681(2)	0.5	0.009(4)
O	4i	0.230(3)	0	0.221(1)	1
O	8j	0.257(2)	0.325(1)	0.2277(8)	1

(F-1)

$S=1.5881$ $R_B=2.708$ $R_F=2.785$ $R_{wp}=11.532$

a=4.9445(5) Å, b=8.5682(5) Å, c=5.0221(2) Å, β=109.030(5)°

atom	site	x	y	z	$occ_{\text{-refined}}$
Li	2c	0	0	0.5	0.991(2)
M	2c	0	0	0.5	0.009(2)
Li	2b	0	0.5	0	0.516(5)
M	2b	0	0.5	0	0.484(5)
Li	4g	0	0.1647(2)	0	0.090(5)
M	4g	0	0.1647(2)	0	0.910(5)
Li	4h	0	0.677(1)	0.5	0.983(6)
M	4h	0	0.677(1)	0.5	0.017(6)
O	4i	0.226(1)	0	0.2177(1)	1
O	8j	0.258(1)	0.3161(5)	0.2286(6)	1

(F-2)

$S=1.6998$ $R_B=3.925$ $R_F=3.343$ $R_{wp}=12.900$

a=4.9459(3) Å, b=8.5663(3) Å, c=5.0224(2) Å, β=109.040(4)°

atom	site	x	y	z	$occ_{\text{-refined}}$
Li	2c	0	0	0.5	0.980(4)
M	2c	0	0	0.5	0.020(4)
Li	2b	0	0.5	0	0.485(5)
M	2b	0	0.5	0	0.515(5)
Li	4g	0	0.1650(2)	0	0.109(5)
M	4g	0	0.1650(2)	0	0.891(5)
Li	4h	0	0.687(1)	0.5	0.977(2)
M	4h	0	0.687(1)	0.5	0.023(6)
O	4i	0.219(1)	0	0.221(1)	1
O	8j	0.259(1)	0.3155(5)	0.2265(6)	1

(G-1)

S=1.9752 R_B=3.145 R_F=3.399 R_{wp}=10.348

a=4.9419(3) Å, b=8.5603(4) Å, c=5.0222(2) Å, β=109.023(4)°

atom	site	x	y	z	occ$_{\text{-refined}}$
Li	2c	0	0	0.5	0.982(3)
M	2c	0	0	0.5	0.018(3)
Li	2b	0	0.5	0	0.498(4)
M	2b	0	0.5	0	0.502(4)
Li	4g	0	0.1647(2)	0	0.040(4)
M	4g	0	0.1647(2)	0	0.960(4)
Li	4h	0	0.677(1)	0.5	0.986(1)
M	4h	0	0.677(1)	0.5	0.014(1)
O	4i	0.222(1)	0	0.216(1)	1
O	8j	0.260(1)	0.3152(4)	0.2286(6)	1

(G-2)

S=1.7254 R_B=5.384 R_F=3.987 R_{wp}=13.203

a=4.9488(4) Å, b=8.5593(5) Å, c=5.0240(2) Å, β=109.013(4)°

atom	site	x	y	z	occ$_{\text{-refined}}$
Li	2c	0	0	0.5	0.988(5)
M	2c	0	0	0.5	0.120(5)
Li	2b	0	0.5	0	0.492(3)
M	2b	0	0.5	0	0.508(3)
Li	4g	0	0.1645(2)	0	0.057(3)
M	4g	0	0.1645(2)	0	0.943(3)
Li	4h	0	0.620(1)	0.5	0.978(3)
M	4h	0	0.620(1)	0.5	0.022(3)
O	4i	0.219(1)	0	0.219(1)	1
O	8j	0.261(1)	0.3153(5)	0.2254(7)	1

(H-1)

S=1.8946 R_B=6.866 R_F=7.029 R_{wp}=15.094

a=4.93891(3) Å, b=8.5486(5) Å, c=5.03268(3) Å, β=109.243(7) °

atom	site	x	y	z	occ$_{\text{-refined}}$
Li	2c	0	0	0.5	0.961(3)
M	2c	0	0	0.5	0.039(3)
Li	2b	0	0.5	0	0.703(7)
M	2b	0	0.5	0	0.257(7)
Li	4g	0	0.1730(5)	0	0.007(7)
M	4g	0	0.1730(5)	0	0.993(7)
Li	4h	0	0.702(1)	0.5	0.986(2)
M	4h	0	0.702(1)	0.5	0.014(2)
O	4i	0.208(2)	0	0.225(1)	1
O	8j	0.257(1)	0.328(1)	0.222(1)	1

(H-2)

S=2.6486 R_B=8.110 R_F=8.745 R_{wp}=18.415

a=4.9321(5) Å, b=8.5369(5) Å, c=5.0241(3) Å, β=109.297.(6) °

atom	site	x	y	z	occ$_{\text{-refined}}$
Li	2c	0	0	0.5	0.930(1)
M	2c	0	0	0.5	0.070(1)
Li	2b	0	0.5	0	0.597(1)
M	2b	0	0.5	0	0.413(1)
Li	4g	0	0.1596(8)	0	0.047(1)
M	4g	0	0.1596(8)	0	0.953(1)
Li	4h	0	0.603(3)	0.5	0.975(5)
M	4h	0	0.603(3)	0.5	0.025(5)
O	4i	0.208(3)	0	0.219(3)	1
O	8j	0.231(2)	0.331(1)	0.216(1)	1

(I-1)

S=2.1849 R_B=7.113 R_F=8.315 R_{wp}=14.453

a=4.9398(2) Å, b=8.5529(2) Å, c=5.03278(3) Å, β=109.224(6) °

atom	site	x	y	z	occ$_{\text{-refined}}$
Li	2c	0	0	0.5	0.970(2)
M	2c	0	0	0.5	0.030(2)
Li	2b	0	0.5	0	0.637(3)
M	2b	0	0.5	0	0.343(3)
Li	4g	0	0.1739(7)	0	0.037(3)
M	4g	0	0.1739(7)	0	0.963(3)
Li	4h	0	0.714(3)	0.5	0.984(2)
M	4h	0	0.714(3)	0.5	0.026(3)
O	4i	0.213(2)	0	0.226(3)	1
O	8j	0.257(2)	0.341(1)	0.219(2)	1

(I-2)

S=2.4442 R_B=13.576 R_F=18.039 R_{wp}=15.653

a=4.9393(4) Å, b=8.5402(2) Å, c=5.0311(3) Å, β=109.2497(5) °

atom	site	x	y	z	occ$_{\text{-refined}}$
Li	2c	0	0	0.5	0.951(2)
M	2c	0	0	0.5	0.049(2)
Li	2b	0	0.5	0	0.532(1)
M	2b	0	0.5	0	0.468(1)
Li	4g	0	0.1733(7)	0	0.044(1)
M	4g	0	0.1733(7)	0	0.956(1)
Li	4h	0	0.718(2)	0.5	0.981(6)
M	4h	0	0.718(2)	0.5	0.019(6)
O	4i	0.219(3)	0	0.222(3)	1
O	8j	0.245(2)	0.346(1)	0.217(1)	1

(J-1)

S=2.591 R_B=8.748 R_F=7.008 R_{wp}=21.632

a=4.9337(5) Å, b=8.5482(5) Å, c=5.0214(4) Å, β=109.0178(7) °

atom	site	x	y	z	occ$_{\text{-refined}}$
Li	2c	0	0	0.5	0.949(3)
M	2c	0	0	0.5	0.051(3)
Li	2b	0	0.5	0	0.481(6)
M	2b	0	0.5	0	0.519(6)
Li	4g	0	0.1619(6)	0	0.073(6)
M	4g	0	0.1619(6)	0	0.927(6)
Li	4h	0	0.681(4)	0.5	0.976(2)
M	4h	0	0.681(4)	0.5	0.024(2)
O	4i	0.218(4)	0	0.207(3)	1
O	8j	0.252(3)	0.314(1)	0.231(1)	1

(J-2)

S=2.335 R_B=6.400 R_F=7.537 R_{wp}=17.704

a=4.9367(2) Å, b=8.5548(3) Å, c=5.0183(2) Å, β=109.035(4) °

atom	site	x	y	z	occ$_{\text{-refined}}$
Li	2c	0	0	0.5	0.963(6)
M	2c	0	0	0.5	0.037(6)
Li	2b	0	0.5	0	0.425(7)
M	2b	0	0.5	0	0.575(7)
Li	4g	0	0.1645(4)	0	0.076(7)
M	4g	0	0.1645(4)	0	0.924(7)
Li	4h	0	0.698(1)	0.5	0.974(3)
M	4h	0	0.698(1)	0.5	0.026(3)
O	4i	0.232(3)	0	0.210(1)	1
O	8j	0.263(2)	0.319(1)	0.225(1)	1

(K-1)

S=1.4651 R_B=4.206 R_F=4.379 R_{wp}=12.996

a=4.9425(2) Å, b=8.5664(3) Å, c=5.0217(2) Å, β=109.059(4) °

atom	site	x	y	z	occ$_{\text{-refined}}$
Li	2c	0	0	0.5	0.951(8)
M	2c	0	0	0.5	0.049(8)
Li	2b	0	0.5	0	0.348(6)
M	2b	0	0.5	0	0.652(6)
Li	4g	0	0.1634(4)	0	0.073(6)
M	4g	0	0.1634(4)	0	0.927(6)
Li	4h	0	0.673(2)	0.5	0.972(5)
M	4h	0	0.673(2)	0.5	0.038(5)
O	4i	0.220(3)	0	0.218(2)	1
O	8j	0.243(3)	0.319(1)	0.225(1)	1

(K-2)

S=1.561 R_B=2.705 R_F=3.344 R_{wp}=11.638

a=4.9425(4) Å, b=8.5664(5) Å, c=5.02173(2) Å, β=109.059(5) °

atom	site	x	y	z	occ$_{\text{-refined}}$
Li	2c	0	0	0.5	0.961(5)
M	2c	0	0	0.5	0.039(5)
Li	2b	0	0.5	0	0.358(3)
M	2b	0	0.5	0	0.642(3)
Li	4g	0	0.1645(3)	0	0.052(3)
M	4g	0	0.1645(3)	0	0.948(3)
Li	4h	0	0.674(2)	0.5	0.988(3)
M	4h	0	0.674(2)	0.5	0.012(3)
O	4i	0.242(2)	0	0.215(1)	1
O	8j	0.253(2)	0.3243(8)	0.2305(7)	1

(L-1)

S=1.7661 R_B=3.607 R_F=4.184 R_{wp}=10.796

a=4.9588(2) Å, b=8.5878(2) Å, c=5.0364(1) Å, β=109.248(3)°

atom	site	x	y	z	occ$_{\text{-refined}}$
Li	2c	0	0	0.5	0.938(7)
M	2c	0	0	0.5	0.062(7)
Li	2b	0	0.5	0	0.266(6)
M	2b	0	0.5	0	0.734(6)
Li	4g	0	0.1636(4)	0	0.065(6)
M	4g	0	0.1636(4)	0	0.935(6)
Li	4h	0	0.670(3)	0.5	0.969(4)
M	4h	0	0.670(3)	0.5	0.031(4)
O	4i	0.217(2)	0	0.211(1)	1
O	8j	0.248(2)	0.324(1)	0.2280(9)	1

(L-2)

S=1.6546 R_B=2.404 R_F=3.333 R_{wp}=9.645

a=4.95636(2) Å, b=8.5793(2) Å, c=5.0279(1) Å, β=109.266(2)°

atom	site	x	y	z	occ$_{\text{-refined}}$
Li	2c	0	0	0.5	0.950(7)
M	2c	0	0	0.5	0.050(7)
Li	2b	0	0.5	0	0.296(5)
M	2b	0	0.5	0	0.704(5)
Li	4g	0	0.1646(4)	0	0.050(5)
M	4g	0	0.1646(4)	0	0.950(5)
Li	4h	0	0.674(2)	0.5	0.981(4)
M	4h	0	0.674(2)	0.5	0.019(6)
O	4i	0.229(2)	0	0.216(1)	1
O	8j	0.252(2)	0.332(1)	0.2275(7)	1

(M-1)

S=1.8144 R_B=3.613 R_F=4.001 R_{wp}=13.727					
a=4.9663(2) Å, b=8.5708(3) Å, c=5.0293(1) Å, β=109.206(3) °					
atom	site	x	y	z	$occ_{-refined}$
Li	2c	0	0	0.5	0.877(7)
M	2c	0	0	0.5	0.123(7)
Li	2b	0	0.5	0	0.169(8)
M	2b	0	0.5	0	0.831(8)
Li	4g	0	0.1650(6)	0	0.087(8)
M	4g	0	0.1650(6)	0	0.913(8)
Li	4h	0	0.656(3)	0.5	0.975(8)
M	4h	0	0.656(3)	0.5	0.025(8)
O	4i	0.227(3)	0	0.228(2)	1
O	8j	0.246(2)	0.323(1)	0.223(1)	1

(M-2)

S=1.7835 R_B=3.319 R_F=3.612 R_{wp}=13.051					
a=4.9661(2) Å, b=8.5709(3) Å, c=5.0293(2) Å, β=109.2057(4) °					
atom	site	x	y	z	$occ_{-refined}$
Li	2c	0	0	0.5	0.933(4)
M	2c	0	0	0.5	0.067(4)
Li	2b	0	0.5	0	0.187(7)
M	2b	0	0.5	0	0.813(7)
Li	4g	0	0.1656(5)	0	0.065(7)
M	4g	0	0.1656(5)	0	0.945(7)
Li	4h	0	0.641(2)	0.5	0.982(5)
M	4h	0	0.641(2)	0.5	0.018(5)
O	4i	0.233(3)	0	0.232(2)	1
O	8j	0.246(2)	0.323(1)	0.219(1)	1

(N-1)

S=1.9049 R_B=3.435 R_F=4.115 R_{wp}=11.764					
a=4.9722(2) Å, b=8.5838(3) Å, c=5.0323(1) Å, β=109.212(3) °					
atom	site	x	y	z	$occ_{-refined}$
Li	2c	0	0	0.5	0.892(6)
M	2c	0	0	0.5	0.108(6)
Li	2b	0	0.5	0	0.227(7)
M	2b	0	0.5	0	0.773(7)
Li	4g	0	0.1646(6)	0	0.113(7)
M	4g	0	0.1646(6)	0	0.887(7)
Li	4h	0	0.667(3)	0.5	0.977(1)
M	4h	0	0.667(3)	0.5	0.023(1)
O	4i	0.231(3)	0	0.216(2)	1
O	8j	0.246(2)	0.3266(5)	0.223(1)	1

(N-2)

S=1.6762 R_B=2.176 R_F=2.415 R_{wp}=10.492					
a=4.9706(2) Å, b=8.6195(3) Å, c=5.0394(1) Å, β=109.116(3) °					
atom	site	x	y	z	$occ_{-refined}$
Li	2c	0	0	0.5	0.948(8)
M	2c	0	0	0.5	0.052(8)
Li	2b	0	0.5	0	0.124(4)
M	2b	0	0.5	0	0.876(4)
Li	4g	0	0.1672(4)	0	0.069(4)
M	4g	0	0.1672(4)	0	0.931(4)
Li	4h	0	0.684(2)	0.5	0.970(5)
M	4h	0	0.684(2)	0.5	0.030(5)
O	4i	0.231(3)	0	0.223(2)	1
O	8j	0.243(2)	0.333(1)	0.226(1)	1

(O-1)

S=1.7518 R_B=4.646 R_F=4.903 R_{wp}=15.551					
a=4.9720(3) Å, b=8.6141(4) Å, c=5.034(2) Å, β=109.145(4) °					
atom	site	x	y	z	$occ_{-refined}$
Li	2c	0	0	0.5	0.888(2)
M	2c	0	0	0.5	0.112(2)
Li	2b	0	0.5	0	0.126(9)
M	2b	0	0.5	0	0.874(9)
Li	4g	0	0.1655(6)	0	0.022(9)
M	4g	0	0.1655(6)	0	0.978(9)
Li	4h	0	0.667(4)	0.5	0.971(1)
M	4h	0	0.667(4)	0.5	0.029(1)
O	4i	0.233(5)	0	0.231(4)	1
O	8j	0.241(4)	0.323(1)	0.220(2)	1

(O-2)

S=1.8947 R_B=3.802 R_F=4.218 R_{wp}=14.410					
a=4.9687(2) Å, b=8.614(3) Å, c=5.0296(1) Å, β=109.1799(4) °					
atom	site	x	y	z	$occ_{-refined}$
Li	2c	0	0	0.5	0.932(5)
M	2c	0	0	0.5	0.068(5)
Li	2b	0	0.5	0	0.076(9)
M	2b	0	0.5	0	0.924(9)
Li	4g	0	0.1670(8)	0	0.049(9)
M	4g	0	0.1670(8)	0	0.951(9)
Li	4h	0	0.667(6)	0.5	0.970(3)
M	4h	0	0.667(6)	0.5	0.030(3)
O	4i	0.228(4)	0	0.234(4)	1
O	8j	0.238(4)	0.326(2)	0.219(2)	1
